# Identification of New Genetic Risk Variants for Type 2 Diabetes

**DOI:** 10.1371/journal.pgen.1001127

**Published:** 2010-09-16

**Authors:** Xiao Ou Shu, Jirong Long, Qiuyin Cai, Lu Qi, Yong-Bing Xiang, Yoon Shin Cho, E. Shyong Tai, Xiangyang Li, Xu Lin, Wong-Ho Chow, Min Jin Go, Mark Seielstad, Wei Bao, Huaixing Li, Marilyn C. Cornelis, Kai Yu, Wanqing Wen, Jiajun Shi, Bok-Ghee Han, Xue Ling Sim, Liegang Liu, Qibin Qi, Hyung-Lae Kim, Daniel P. K. Ng, Jong-Young Lee, Young Jin Kim, Chun Li, Yu-Tang Gao, Wei Zheng, Frank B. Hu

**Affiliations:** 1 Division of Epidemiology, Department of Medicine, Vanderbilt Epidemiology Center, Vanderbilt-Ingram Cancer Center, Vanderbilt University School of Medicine, Nashville, Tennessee, United States of America; 2 Departments of Epidemiology and Nutrition, Harvard University School of Public Health, Boston, Massachusetts, United States of America; 3 Department of Epidemiology, Shanghai Cancer Institute, Shanghai, China; 4 Center for Genome Science, Korea National Institute of Health, Seoul, Korea; 5 Department of Medicine, Yong Loo Lin School of Medicine, National University of Singapore, Singapore, Singapore; 6 Department of Epidemiology and Public Health, Yong Loo Lin School of Medicine, National University of Singapore, Singapore, Singapore; 7 Duke-National University of Singapore Graduate Medical School, Singapore, Singapore; 8 Department of Nutrition and Food Hygiene and MOE Key Lab of Environment and Health, School of Public Health, Tongji Medical College, Huazhong University of Science and Technology, Wuhan, China; 9 Key Laboratory of Nutrition and Metabolism, Institute for Nutritional Sciences, Shanghai Institutes for Biological Sciences, Chinese Academy of Sciences and Graduate School of the Chinese Academy of Sciences, Shanghai, China; 10 Division of Cancer Epidemiology and Genetics, National Cancer Institute, Rockville, Maryland, United States of America; 11 Genome Institute of Singapore, Singapore, Singapore; 12 Department of Biostatistics, Vanderbilt University School of Medicine, Nashville, Tennessee, United States of America; Queensland Institute of Medical Research, Australia

## Abstract

Although more than 20 genetic susceptibility loci have been reported for type 2 diabetes (T2D), most reported variants have small to moderate effects and account for only a small proportion of the heritability of T2D, suggesting that the majority of inter-person genetic variation in this disease remains to be determined. We conducted a multistage, genome-wide association study (GWAS) within the Asian Consortium of Diabetes to search for T2D susceptibility markers. From 590,887 SNPs genotyped in 1,019 T2D cases and 1,710 controls selected from Chinese women in Shanghai, we selected the top 2,100 SNPs that were not in linkage disequilibrium (r^2^<0.2) with known T2D loci for *in silico* replication in three T2D GWAS conducted among European Americans, Koreans, and Singapore Chinese. The 5 most promising SNPs were genotyped in an independent set of 1,645 cases and 1,649 controls from Shanghai, and 4 of them were further genotyped in 1,487 cases and 3,316 controls from 2 additional Chinese studies. Consistent associations across all studies were found for rs1359790 (13q31.1), rs10906115 (10p13), and rs1436955 (15q22.2) with *P*-values (per allele OR, 95%CI) of 6.49×10^−9^ (1.15, 1.10–1.20), 1.45×10^−8^ (1.13, 1.08–1.18), and 7.14×10^−7^ (1.13, 1.08–1.19), respectively, in combined analyses of 9,794 cases and 14,615 controls. Our study provides strong evidence for a novel T2D susceptibility locus at 13q31.1 and the presence of new independent risk variants near regions (10p13 and 15q22.2) reported by previous GWAS.

## Author Summary

Type 2 diabetes, a complex disease affecting more than a billion people worldwide, is believed to be caused by both environmental and genetic factors. Although some studies have shown that certain genes may make some people more susceptible to type 2 diabetes than others, the genes reported to date have only a small effect and account for a small proportion of type 2 diabetes cases. Furthermore, few of these studies have been conducted in Asian populations, although Asians are known to be more susceptible to insulin resistance than people living in Western countries, and incidence of type 2 diabetes has been increasing alarmingly in Asian countries. We conducted a multi-stage study involving 9,794 type 2 diabetes cases and 14,615 controls, predominantly Asians, to discover genes related to susceptibility to type 2 diabetes. We identified 3 genetic regions that are related to increased risk of type 2 diabetes.

## Introduction

Type 2 diabetes (T2D) is a common complex disease that affects over a billion people worldwide [1]. Through genome-wide association studies (GWAS), at least 24 genetic susceptibility loci have been reported for T2D [1]–[9], including a SNP, rs7593730, at 2q24 near the RBMS1 and ITGB6 genes that was associated with diabetes risk in a recent report from the Nurses' Health Study/Health Professionals Follow-up Study (NHS/HPFS) [2]. However, most of the reported genetic variants have small to moderate effects and account for only a small proportion of the heritability of T2D, suggesting that the majority of inter-person genetic variation in this disease remains to be determined. Over the last two decades, China, like many other Asian countries, has experienced a dramatic increase in T2D incidence. Cumulative evidence suggests that Asians may be more susceptible to insulin resistance compared with populations of European ancestry [10]. However, among the previously reported T2D genetic markers, only three SNPs – including two reported very recently – have been identified in populations of Asian ancestry [8], [9]. SNP rs2283228 in the *KCNQ1* gene was identified in a 3-stage study that included 194 diabetes patients and 1,558 controls and 268,068 SNPs in the first (discovery) stage [8]. A study conducted among Han Chinese in Taiwan recently identified two additional novel loci in the protein tyrosine phosphatase receptor type D (*PTPRD*; *P* = 8.54×10^−10^) and serine racemase (*SRR*; *P* = 3.06×10^−9^) genes [9].

Large genetic studies conducted in Asian populations will facilitate the identification of additional genetic markers for T2D, particularly for markers with a higher frequency in Asians than in other populations. We recently completed a GWAS of T2D in Shanghai. We report here our first effort, using a fast-track, multiple-stage study approach, to identify novel genetic markers for diabetes.

## Methods

### Ethics statement

The study protocol was approved by the institutional review boards at Vanderbilt University Medical Center and at each of the collaborating institutes. Informed consent was obtained from all participants.

### Study design and population

This study consisted of a discovery stage and two validation stages, i.e. an *in silico* and a *de novo* validation study. The overall study design is presented in Figure S1.

The discovery stage included 1,019 T2D cases, 886 incident T2D cases from the Shanghai Women's Health Study (SWHS), an ongoing, population-based, prospective cohort study of women living in Shanghai, and 133 prevalent T2D cases identified among controls of the Shanghai Breast Cancer Study (SBCS), who were recruited in Shanghai during approximately the same period as the SWHS [11]. Controls for the discovery phase were 1,710 non-diabetic female controls from the SBCS (for further details, see Text S1, online). The biologic samples used for genotyping in this study were collected by the SWHS and SBCS.

### Genotyping and quality control procedures

DNA samples were genotyped using the Affymetrix Genome-Wide Human SNP Array 6.0. Extensive quality control (QC) procedures were implemented in the study. In the SWHS/SBCS GWAS scan, three positive QC samples purchased from Coriell Cell Repositories and a negative QC sample were included in each of the 96-well plates of the Affymetrix SNP Array 6.0. SNP data obtained from positive quality control samples showed a very high concordance rate of called genotypes based on 79,764,872 comparisons (mean, 99.87%; median, 100%). Samples with genotyping call rates less than 95% were excluded. The sex of all study samples was confirmed to be female. The identity-by-descent analysis based on identity by state was performed to detect first-degree cryptic relationships using PLINK version 1.06 [12]. We excluded from the study 21 samples that had: 1) call rate <95% (n = 5); 2) samples that were contaminated or had mixed-up labels or that had been duplicated (n = 12); 3) first-degree relatives, such as parent-offspring or full siblings (n = 4).

We also excluded from the analysis SNPs that met any of following criteria: 1) MAF <0.05; 2) call rate <95%; 3) P for Hardy-Weinburg equilibrium HWE <0.00001 in either the case or control groups or in the combined data set; 4) concordance rate <95% among the duplicated QC samples; 5) significant difference in allele frequency distribution (P<0.00001) between the 886 T2D cases from the SWHS and the 133 T2D cases from the SBCS; 6) significant difference in missing rates between cases and controls (P<0.00001). After applying the QC filter, 590,887 SNPs remained for the analyses.

Because of financial constraints, we conducted a fast-track validation study using an approach that combined *in silico* and *de novo* replication. We selected a total of 2,100 SNPs from the discovery phase that had P-values of 1.3×10^−9^ to 5.0×10^−3^ derived from the additive model and that were not in linkage disequilibrium (LD; r^2^<0.2 based on the HapMap CHB dataset) with any previously reported T2D GWAS SNPs for an *in silico* replication using the GWAS scan data from the NHS/HPFS [2]. We used the NHS/HPFS T2D GWAS scans for our first step of validation, because the Shanghai T2D GWAS was conducted concurrently and used the same genotyping platform as the NHS/HPFS T2D GWAS and *a priori* arrangement was made for the two studies to exchange the top 2,000 SNPs for *in silico* replication. The NHS/HPFS T2D GWAS included 2,591 cases and 3,052 controls of European ancestry. We recognize that this approach may have reduced our chances of finding ethnicity-specific T2D markers, however, this approach had the advantage of enhancing our ability of finding true genetic markers. From the first *in silico* replication, 65 SNPs with the same direction of association in both studies and with a MAF >20% were chosen for a second *in silico* replication using GWAS scan data from a Korean T2D study, which included 1,042 cases and 2,943 controls genotyped with the Affymetrix Genome-Wide Human SNP Array 5.0 platform. In order to improve yield, only the top SNPs that are included in Affymetrix 5.0 (N = 56) or that are in high LD (r^2^>0.8) with at least one SNP on Affymetrix 5.0 (N = 9) were selected for replication (Table S1). Of the 65 SNPs, the top 8 SNPs replicated in the Korean T2D study were further investigated using GWAS data from a T2D study conducted among Singapore Chinese (2,010 cases and 1,945 controls) who were genotyped by using Illumina HumanHap 610 or Illumina Human1M (Table S2). Four of the 8 SNPs were not directly genotyped in the Singapore study, so instead, we selected SNPs that are in strong LD with these 4 SNPs (imputed SNP information became available recently and is presented in this report). Finally, the 5 top SNPs (rs2815429, rs10906115, rs1359790, rs10751301, and rs1436955) were selected for *de novo* genotyping in an independent sample set of 1,645 T2D cases and 1,649 controls identified from the SWHS and Shanghai Men's Health Study (SMHS). Four of these SNPs (rs10906115, rs1359790, rs10751301, and rs1436955) were selected for the final stage of *de novo* genotyping replication in two independent Chinese studies, the Wuhan Diabetes Study (WDS; 1,063 cases and 1,408 controls) and the Nutrition and Health of Aging Population in China (NHAPC) study (424 cases and 1,908 controls). Detailed descriptions of the study designs and populations for each of the participating studies are presented in Text S1 online.

Genotyping for the 5 SNPs included in the SWHS and SMHS sample set was completed using the iPLEX Sequenom MassArray platform. Included in each 96-well plate as quality control samples were two negative controls, two blinded duplicates, and two samples included in the HapMap project. We also included 65 subjects who had been genotyped by the Affymetrix SNP Array 6.0 in the Sequenom genotyping. The consistency rate was 100% for all SNPs for the blinded duplicates, compared with the HapMap data and compared with data from the Affymetrix SNP Array 6.0. Genotyping for the final 4 SNPs in the WDS and NHAPC was completed using TaqMan assays at the two local institute laboratories using reagents provided by the Vanderbilt Molecular Epidemiology Laboratory. Both laboratories were asked to genotype a trial plate provided by the Vanderbilt Molecular Epidemiology Laboratory that contained DNA from 70 Chinese samples before the main study genotyping was conducted. The consistency rates for these trial samples were 100% compared with genotypes previously determined at Vanderbilt for all four SNPs in both local laboratories. In addition, replicate samples comparing 3–7% of all study samples were dispersed among genotyping plates for both studies.

### Imputation

The imputation of un-genotyped SNPs in all participating GWASs was carried out after the completion of the current study using the programs MACH (http://www.sph.umich.edu/csg/abecasis/MACH/) or IMPUTE (https://mathgen.stats.ox.ac.uk/impute) with HapMap Asian data as the reference for Asians and CEU data as the reference for European-ancestry samples. Only data with high imputation quality (RSQR >0.3 for MACH) were included in the current analysis.

### Statistical analyses

PLINK version 1.06 was used to analyze genome-wide data obtained in the SBCS/SWHS GWAS scan. Population structure was evaluated by principal component analysis using EIGENSTRAT (http://genepath.med.harvard.edu/~reich/Software.htm).A set of 12,533 SNPs with a MAF ≥10% in Chinese samples and a distance of ≥25 kb between two adjacent SNPs was selected to evaluate the population structure. The first two principal components were included in the logistic regression models for adjustment of population structures. The inflation factor λ was estimated to be 1.03, suggesting that population substructure, if present, should not have any appreciable effect on the results.

Pooled and meta-analyses were carried out in SAS to derive combined odds ratios (OR) by using data from studies of all stages. We applied the weighted z-statistics method, where weights are proportional to the square root of the number of subjects in each study. Results from both random and fixed effect models are presented.

ORs and 95% confidence intervals (CI) were estimated using logistic regression models with adjustment for age, BMI, population structure (for GWAS data), and gender, when appropriate. Analyses with additional adjustment for smoking were conducted by pooled analysis whenever possible and by meta-analysis when KARE data were included in order to examine the confounding and modification effects of these factors (Table S2). Genotype distributions for the top 4 SNPs included in the final *de novo* genotyping were consistent with HWE (P> 0.05) in each study. All P values presented are based on two-tailed tests, except where indicated otherwise.

## Results

The general characteristics of the participating study populations are presented in Table 1. T2D cases had a higher BMI than controls across all studies. Except for the SWHS, SMHS, and Shanghai Nutrition Institute (SNI) validation studies, where cases and controls were matched on age, cases were older than controls in all other studies. A difference in gender distribution was also seen in several studies. These variables were adjusted for in subsequent analyses.

**Table 1:** Characteristics of the study participants.

Study population			Cases	Controls	% Men		Age^a^		BMI^a^	
		Study area	N = 9,794	N = 14,615	Cases	Controls	Cases	Controls	Cases	Controls
**Discovery Stage**	SBCS/SWHS	Shanghai^b^	1019	1710	0.0	0.0	51.7±6.7	48.7±8.5	26.5±3.7	23.1±3.3
**Replication Set I**	NHS/HPFS	USA	2591	3052	43.4	42.5	54.2±7.6	53.9±7.5	27.6±4.6	24.1±3.6
	KARE	Korea	1042	2943	51.7	46.0	56.4±8.6	51.1±8.6	25.5±3.3	24.1±2.9
	SDCS/SP2	Singapore^c^	2010	1945	50.0	42.0	64.4±10.2	47.2±10.7	25.3±3.9	22.6±3.6
**Replication Set II**	SWHS/SMHS	Shanghai^b^	1645	1649	44.6	44.5	58.9±8.8	58.9±8.7	26.3±3.5	24.2±3.4
	NHAPC	Shanghai/Beijing b	424	1908	48.8	41.5	59.7±5.7	58.4±6.0	25.4±3.5	23.8±3.4
	WDS	Wuhan^b^	1063	1408	57.6	58.7	50.7±10.4	42.8±10.0	24.8±3.7	23.0±3.1

a Age and BMI are presented as mean ± SD.

b Conducted in China and included Chinese participants only.

c Only Chinese participants were included.

Table 2 presents the results of analyses of associations of T2D with previously reported, GWAS-identified genetic markers in our discovery samples [1]–[9]. Of the 24 SNPs reported by previous GWAS, 15 were directly genotyped by the Affymetrix SNP Array 6.0. One SNP (rs7578597) showed a MAF = 0 in HapMap CHB data and was not included on the Affymetrix 6.0 chip. The remaining 8 SNPs, including rs2943641, rs10010131, rs13266634, rs12779790, and rs4430796, as well as the newly identified markers rs391300 and rs17584499, were imputed. SNP rs4430796 showed low imputation quality (RSQR = 0.06) in the SBCS/SWHS GWAS and was excluded from the analysis.

**Table 2:** Associations of previously-reported T2D related SNPs with disease risk in Chinese women.

					Risk allele frequency					
SNP^a^	Region	Alleles^b^	Gene	Genotyping^c^	Cases	Controls	OR (95% CI)^d^	P for trend^e^	Reported Effect	Power to detect association^f^
rs10923931 [1]	1p12	T/G	NOTCH2,ADAM30	Affy 6.0	0.03	0.03	1.02 (0.71–1.48)	0.454	1.13 (1.08–1.17)	0.19
rs2943641 [3]	2q36.3	C/T	LOC64673/IRS1	Imputed	0.93	0.93	1.20 (0.94–1.54)	0.070	1.19 (1.13–1.25)	0.45
rs1801282 [1]	3p25.2	C/G	PPARG	Affy 6.0	0.94	0.95	0.99 (0.76–1.30)	0.523	1.14 (1.08–1.20)	0.25
rs4607103 [1]	3p14.1	C/T	ADAMTS9	Affy 6.0	0.62	0.63	1.03 (0.91–1.17)	0.302	1.09 (1.06–1.12)	0.43
rs4402960 [6]	3q27.2	T/G	IGF2BP2	Affy 6.0	0.28	0.24	1.28 (1.12–1.49)	2.4×10^−4^	1.14 (1.11–1.18)	0.65
rs10010131 [4]	4p16.1	G/A	WFS1	Imputed	0.95	0.95	0.95 (0.71–1.28)	0.632	1.11 (1.05–1.16)	0.19
rs10946398 [6]	6p22.3	C/A	CDKAL1	Affy 6.0	0.45	0.41	1.20 (1.06–1.36)	0.002	1.14 (1.11–1.17)	0.75
rs864745 [1]	7p15.1	T/C	JAZF1	Affy 6.0	0.78	0.78	0.97 (0.84–1.13)	0.637	1.10 (1.07–1.13)	0.40
rs13266634 [7]	8q24.11	C/T	SLC30A8	Imputed	0.61	0.58	1.19 (1.04–1.35)	0.004	1.15 (1.12–1.19)	0.79
rs10811661 [6]	9p21.3	T/C	CDKN2A/B	Affy 6.0	0.56	0.52	1.24 (1.10–1.40)	3.05×10^−4^	1.20 (1.14–1.25)	0.95
rs564398 [5]	9p21.3	T/C	CDKN2B	Affy 6.0	0.89	0.88	1.08 (0.89–1.31)	0.209	1.12 (1.07–1.17)	0.36
rs12779790 [1]	10p13	G/A	CDC123,CAMK1D	Imputed	0.17	0.17	1.04 (0.88–1.23)	0.309	1.11 (1.07–1.14)	0.42
rs5015480 [1]	10q23.33	C/T	HHEX	Affy 6.0	0.20	0.17	1.42 (1.20–1.66)	9.1×10^−6^	1.17 (1.11–1.24)	0.70
rs7901695 [7]	10q25.2	C/T	TCF7L2	Affy 6.0	0.04	0.03	1.41 (1.03–1.93)	0.017	1.37 (1.31–1.43)	0.67
rs2283228 [8]	11p15.5	C/T	KCNQ1	Affy 6.0	0.66	0.62	1.16 (1.03–1.30)	0.003	1,26 (1.18–1.34)	0.96
rs5215 [4]	11p15.1	C/T	KCNJ11	Affy 6.0	0.42	0.38	1.21 (1.07–1.37)	0.001	1.14 (1.10–1.19)	0.74
rs1495377 [5]	12q15	G/C	NR	Affy 6.0	0.27	0.27	0.97 (0.84–1.11)	0.683	1.12 (1.06–1.18)	0.57
rs7961581 [1]	12q21.1	C/T	TSPAN8,LGR5	Affy 6.0	0.20	0.21	0.96 (0.82–1.12)	0.708	1.09 (1.06–1.12)	0.36
rs8050136 [6]	16q12.2	A/C	FTO	Affy 6.0	0.13	0.12	0.98 (0.81–1.19)	0.574	1.17 (1.12–1.22)	0.60
rs391300 [9]	17p13.3	C/T	SRR	Imputed	0.71	0.72	0.96 (0.83–1.10)	0.739	1.28 (1.18–1.39)	0.99
rs17584499 [9]	9p24.1	T/C	PTPRD	Imputed	0.10	0.10	0.97 (0.71–1.32)	0.575	1.57 (1.13–1.83)	1.00
rs7593730 [2]	2q24.2	T/C	RBMS1/ITGB6	Affy 6.0	0.82	0.84	0.96 (0.82–1.14)	0.331	1.11 (1.08–1.16)	0.40

a Two SNPs were not included in the current analysis because of poor imputation quality (RSQR = 0.06 for rs4430796) or a very low MAF in the Chinese population (rs7578597 MAF = 0 in HapMap CHB samples).

b Risk allele/reference allele initially reported (based on forward strand).

c For imputed SNPs, dosage data with imputation uncertainty taken into account were used to evaluate the association.

d Results were derived from analysis of 1,019 cases and 1,710 controls with adjustment for age, BMI, and two major principal components.

e One-tailed p-values.

f The power was estimated under the additive model and given the reported effect size, 1,019 cases, 1,710 controls, and α = 0.05 (one-sided).

We found that 8 of these SNPs showed an association consistent with initial reports at P<0.05, including rs4402960 (3q27.2, IGF2BP2), rs10946398 (6p22.3, CDKAL1), rs13266634 (8q24.11, SLC30A8), rs10811661 (9p21.3, CDKN2A/B), rs5015480 (10q23.33, HHEX), rs7901695 (10q25.2, TCF7L2), rs2283228 (11p15.5, KCNQ1), and rs5215 (11p15.1, KCNJ11). Among the remaining 11 SNPs, 4 SNPs had a MAF of 3–7% in our study population. Thus, our study did not have sufficient statistical power (statistical power range: 19–45%) to replicate these markers (Table 2). Associations of T2D with SNPs that are in LD with the reported T2D SNPs discovered in European-ancestry populations or in Asians are presented in Table S3.

**Figure 1:**
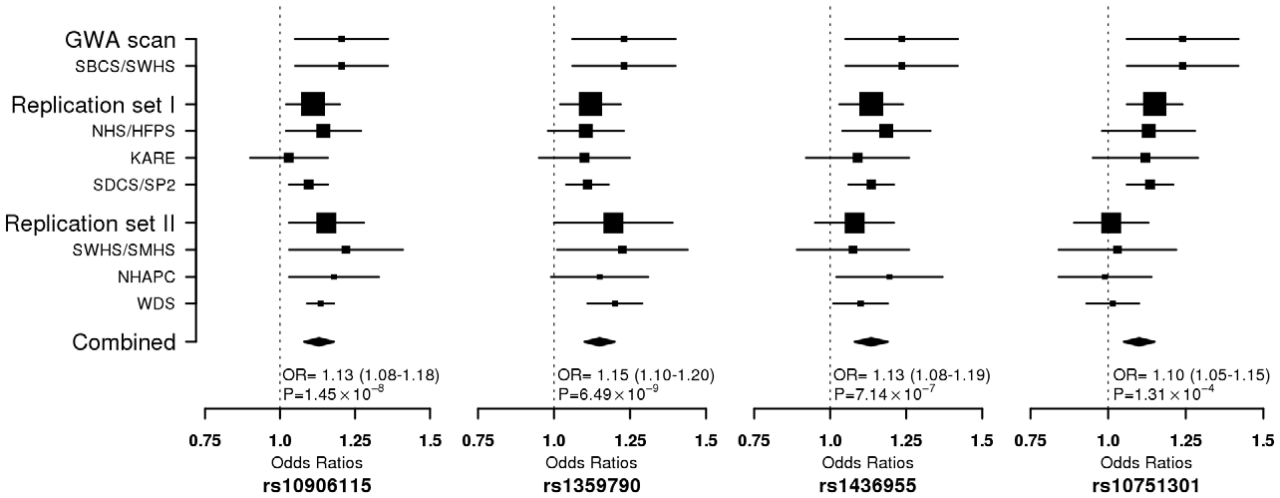
Forest plot for per-allele ORs for the association of T2D risk with 4 SNPs in all participating studies.

Multidimensional scaling analyses of the GWAS scan data showed no evidence of apparent genetic admixture in our study population (Figure S2). The observed number of SNPs with a small P value was larger than expected by chance (Figure S3). We found that rs10906115 (10p13), rs1359790 (13q31.1), and rs1436955 (15q22.2) were consistently associated with T2D across all studies, although the 95% CI for the per allele ORs in several studies included 1.0 (Table 3; Figure 1). *P*-values for trend tests (per allele OR, 95% CI) from meta-analyses of data from all studies were highly statistically significant for these associations: 1.45×10^−8^ for rs10906115 (1.13, 1.08–1.18), 6.49×10^−9^ for rs1359790 (1.15, 1.10–1.20), and 7.14×10^−7^ for rs1436955 (1.13, 1.08–1.19). These *P*-values were below (for rs1359790 and rs10906115) or near (for rs1436955) the genome-wide significance level of 5.0×10^−8^. SNP rs10751301 (11q14.1) was not replicated in the Singapore or *de novo* genotyping studies; the *P*-value for the meta-analysis was 1.31×10^−4^ in the fixed effect model and 0.004 in the random effect model. Additional adjustment for smoking history did not appreciably change the point estimates described above, although the *P*-values were slightly elevated (Table S2).

**Table 3:** Associations of T2D risk with the top four SNPs by study phase.

				Number		Frequency^b^		OR(95% CI)^c^	
SNP	Region	Alleles^a^	Study Set	Cases	Controls	Cases	Controls	Per allele	P for trend
rs10906115	10p13	**A**/G	GWA scan	1019	1710	0.65	0.62	1.20 (1.05–1.36)	0.007
			Replication Set I d	5613	7918	0.60	0.57	1.10 (1.03–1.16)	0.002
			Replication Set II	3115	4944	0.65	0.62	1.17 (1.09–1.18)	1.95×10^−5^
			Fixed model					1.13 (1.08–1.18)	1.45×10^−8^
			Random model					1.13 (1.08–1.18)	1.45×10^−8^
			Homogeneity test					P = 0.618	
rs1359790	13q31.1	**G**/A	GWA scan	1009	1690	0.75	0.71	1.22 (1.06–1.40)	0.006
			Replication Set I^d^	5604	7864	0.73	0.71	1.10 (1.04–1.18)	0.002
			Replication Set II	3117	4907	0.74	0.71	1.20 (1.11–1.29)	6.19×10^−6^
			Fixed model					1.15 (1.10–1.20)	6.49×10^−9^
			Random model					1.15 (1.10–1.20)	6.49×10^−9^
			Homogeneity test					P = 0.665	
rs1436955	15q22.2	**C**/T	GWA scan	1019	1709	0.79	0.75	1.22 (1.05–1.42)	0.008
			Replication Set I d	5642	7938	0.77	0.73	1.13 (1.06–1.21)	2.41×10^−4^
			Replication Set II	3126	4944	0.78	0.76	1.10 (1.01–1.19)	0.024
			Fixed model					1.13 (1.08–1.19)	7.14×10^−7^
			Random model					1.13 (1.08–1.19)	7.14×10^−7^
			Homogeneity test					0.739	
rs10751301	11q14.1	**C**/G	GWA scan	1018	1710	0.24	0.21	1.23 (1.06–1.42)	0.006
			Replication Set I d	5536	7579	0.37	0.33	1.13 (1.06–1.21)	9.09×10^−5^
			Replication Set II	3123	4938	0.22	0.21	1.01 (0.93–1.10)	0.863
			Fixed model					1.10 (1.05–1.15)	1.31×10^−4^
			Random model					1.09 (1.03–1.16)	0.004
			Homogeneity test					P = 0.199	

Notes:

a Bold alleles are risk alleles.

b Frequency of risk alleles.

c Adjusted for age, gender, BMI, two major principal components, and study site when appropriate.

d Derived from meta-analysis.

In an exploratory analysis stratified by smoking, BMI, family history of T2D, and age at diagnosis, SNP rs1359790 showed a slightly stronger association with T2D risk among non-smokers (per allele OR = 1.19, 95% CI = 1.12–1.26, P = 6.4×10^−8^) than among smokers (OR = 1.09, 95% CI = 1.00–1.19, P = 0.044) with a P value of 0.11 for interaction (Table S4). None of the SNPs were related to age at onset of T2D. Neither family history of T2D nor BMI altered the SNP-T2D associations under study.

## Discussion

Using the GWAS data from our discovery stage samples, we were able to validate 8 of 22 previously reported, GWAS-identified T2D SNPs, lending strong support to the validity of the initial discovery samples and methodologies. Applying a fast-track validation study approach, we also identified three promising new T2D markers.

**Figure 2:**
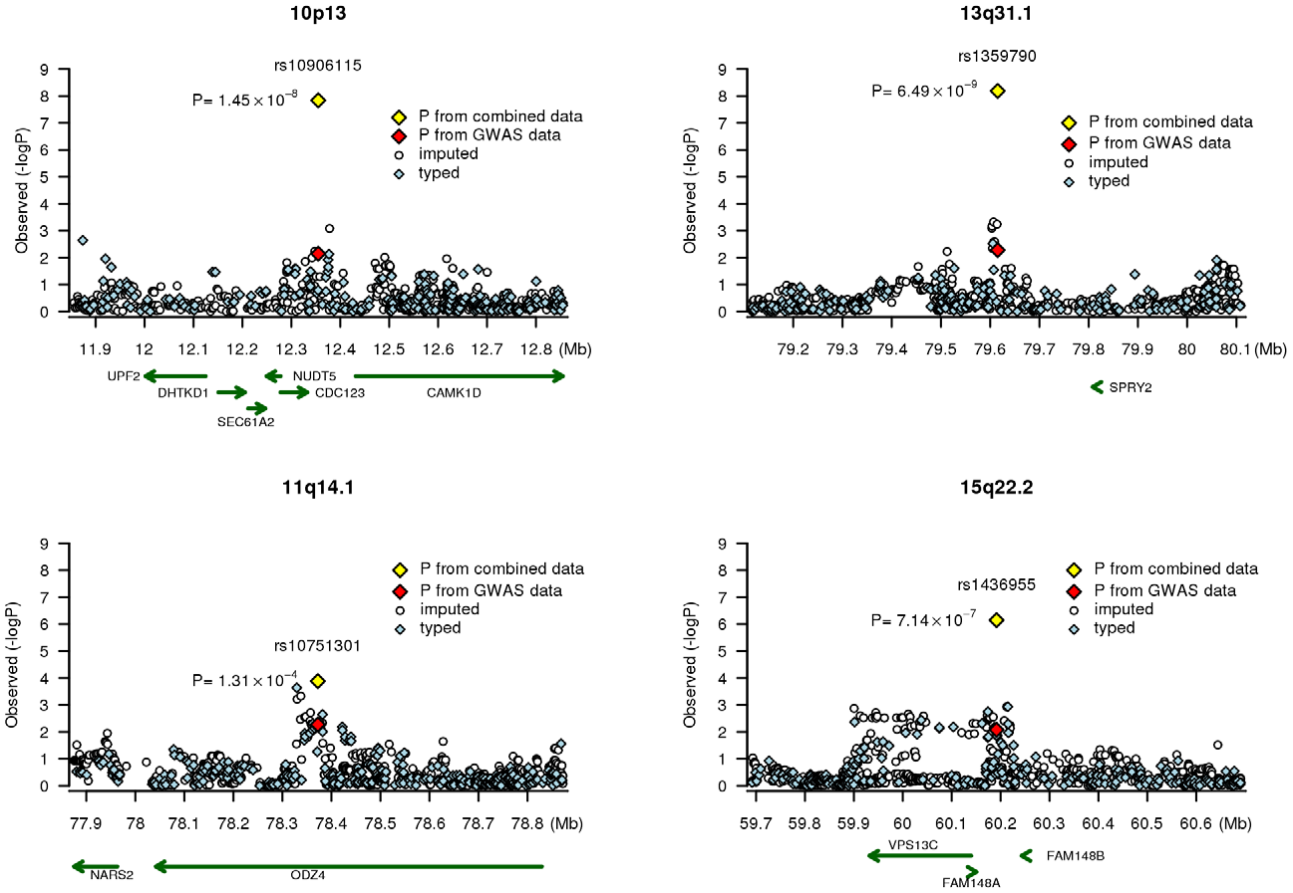
Association signals at four chromosome regions. Results (-logP) are shown for directly genotyped (blue diamonds) and imputed (white circles) SNPs for a 1Mb region centered on the SNP of interest. Gene locations are from the March 2006 UCSC genome browser assembly.

The most significant association identified by our study was for rs1359790 (13q13.1), a novel genetic susceptibility locus identified for T2D (Figure 2). Several transcription factors, such as NIT-2, CdxA, GATA-2, and CDP, bind to this polymorphic site. The C to T transition eliminates a GATA-2 binding site and creates a TATA binding site. The closest known gene, sprouty homolog 2 (Drosophila) (*SPRY2*), is located 193 kb upstream of rs1359790. The *SPRY2* gene encodes a protein belonging to the sprouty family and inhibits growth factor-mediated, receptor tyrosine kinase-induced, mitogen-activated protein kinase signaling [13]. The encoded protein contains a carboxyl-terminal cysteine-rich domain essential for the inhibitory activity of receptor tyrosine kinase signaling proteins and is required for growth factor-stimulated translocation of the protein to membrane ruffles [13], [14]. SPRY2 also modulates the apoptotic actions induced by the pro-inflammatory cytokine, tumor necrosis factor-alpha [15]. SPRY4, a homolog of SPRY2, inhibits the insulin receptor-transduced MAPK signaling pathway [16] and regulates development of the pancreas [17].

SNP rs10906115 is located on chromosome 10p13 (Figure 2), 13.0 kb from rs12779790, which was reported by a previous GWAS of T2D [1]. These two SNPs, however, are in low LD in both Chinese (r^2^ = 0.06) and European populations (r^2^ = 0.19) based on HapMap data. SNP rs12779790 was not included in the Affymetrix SNP Array 6.0, Illumina HumanHap 610-Quad, or Human1M-Duo; thus, it was imputed for both the SBCS/SWHS and the NHS/HPFS by using MACH with RSQR>0.9 and for the Singapore studies using IMPUTE with PROPER_INFO >0.85. The imputed SNP rs12779790 was associated with a per allele OR of 1.10 (95% CI = 1.01–1.19, P = 0.035) in the analysis of pooled data from three studies. However, when both rs12779790 and rs10906115 were included in the same logistic model, the association with rs10906115 remained statistically significant (per allele OR = 1.09, 95% CI = 1.02–1.16, P = 0.007), while the association with rs12779790 was no longer statistically significant (per allele OR = 1.04 [95% CI = 0.96–1.12], P = 0.38; Table 4). These data provide strong evidence that rs10906115 is a new genetic variant at 10p13 independent of the previously-identified SNP rs12779790.

**Table 4:** Association of SNPs rs10906115 and rs12779790 with type 2 diabetes.

SNP		Study	OR (95% CI)^a^	P
rs10906115	Without adjustment for rs12779790	SBCS/SWHS	1.20 (1.05–1.36)	0.007
		NHS/HPFS	1.10 (1.02–1.20)	0.020
		Singapore	1.02 (0.90–1.16)	0.74
		Combined^b^	1.10 (1.04–1.17)	5.0×10^−4^
	With adjustment for rs12779790	SBCS/SWHS	1.21 (1.05–1.38)	0.007
		NHS/HPFS	1.07 (0.98–1.17)	0.16
		Singapore	1.01 (0.88–1.16)	0.86
		Combined^b^	1.09 (1.02–1.16)	0.007
rs12779790	Without adjustment for rs10906115	SBCS/SWHS	1.05 (0.90–1.23)	0.54
		NHS/HPFS	1.16 (1.04–1.28)	0.006
		Singapore	1.04 (0.89–1.23)	0.63
		Combined^b^	1.10 (1.01–1.19)	0.035
	With adjustment for rs10906115	SBCS/SWHS	0.97 (0.82–1.15)	0.73
		NHS/HPFS	1.12 (1.01–1.25)	0.048
		Singapore	1.04 (0.87–1.23)	0.69
		Combined^b^	1.04 (0.96–1.12)	0.38

a Adjusted for age, gender, BMI, and two major principal components.

b Derived from meta-analysis.

SNP rs10906115 is located 22.4 kb downstream of the cell division-cycle 123 homolog (*S. cerevisiae)* (*CDC123)* gene and 76.6 kb upstream of the calcium/calmodulin-dependent protein kinase ID *(CAMK1D)* gene (Figure 2). The *CDC123* gene encodes a protein involved in cell cycle regulation and nutritional control of gene transcription [18]. The *CAMK1D* gene encodes a member of the Ca2+/calmodulin-dependent protein kinase 1 subfamily of serine/threonine kinases. The encoded protein may be involved in the regulation of granulocyte function through the chemokine signal transduction pathway [19]. The role of the *CDC123* and *CAMK1D* genes in the etiology of T2D is unclear.

SNP rs1436955, located on chromosome 15q22.2 (Figure 2), is 51.4 kb downstream of a C2 calcium-dependent domain containing the 4B gene *(C2CD4B;* also known as *NLF2* or *FAM148B*). *C2CD4B* is up-regulated by pro-inflammatory cytokines and may play a role in regulating genes that control cellular architecture [20]. The role of inflammation in the pathophsyiology of T2D has been suggested previously [21]–[25]. *C2CD4B* and *SPRY2* are both highly expressed in human pancreatic tissue [26]. Intriguingly, a very recent report from the Meta-Analysis of Glucose and Insulin-related traits Consortium (MAGIC) found that a SNP (rs11071657) near the *C2CD4B* gene was associated with fasting glucose (P = 3.6×10^−8^) and T2D (P = 2.9×10^−3^) [27]. SNPs rs11071657 and rs1436955, however, are not in LD (r^2^ = 0.04) in Asians, although they are weakly related (r^2^ = 0.25) in Europeans, according to HapMap data. SNP rs11071657 is not included in the Affymetrix SNP 6.0 array. Imputed data from the SBCS/SWHS GWAS showed that this SNP was not significantly associated with T2D risk (per A allele OR = 1.06, 95% CI = 0.94–1.19), although the direction of the association was consistent with that reported by the MAGIC consortium [27]. Adjusting for rs11071657 did not alter the association of T2D risk with rs1436955 (per allele OR = 1.21, 95% CI = 1.06–1.39, P = 0.006). Again, these data strongly imply that rs1436955 may be a new genetic risk variant for T2D at 15q22.2 independent of the recently reported SNP rs11071657.

In summary, in this first GWAS of T2D conducted in a Chinese population, we identified a novel genetic susceptibility locus for T2D, rs1359790, at 13q31.1. Furthermore, we revealed two new genetic variants (rs10906115 at 10p13 and rs1436955 at15q22.2) near T2D susceptibility loci previously reported by GWAS of T2D conducted in European-ancestry populations. Our study demonstrates the value of conducting GWAS in non-European populations for the identification of novel genetic susceptibility markers for T2D.

## Supporting Information

Table S1 Association of 65 SNPs included in Replication Set I with T2D risk.
(0.13 MB DOC)

Table S2 Association of the top 4 SNPs with T2D risk with additional adjustments.
(0.07 MB DOC)

Table S3 Association of SNPs that are in LD with established SNPs in European-ancestry populations (for SNPs discovered in European ancestry populations) or with the reported SNPs in Asians (for SNPs discovered in Asians).
(0.48 MB DOC)

Table S4 Results for the four SNPs showing promising associations with T2D stratified by smoking, BMI, family history of type 2 diabetes, and age of diagnosis in the combined data.
(0.07 MB DOC)

Figure S1 Study design.
(0.09 MB TIF)

Figure S2 MDS analyses to confirm all subjects were Asians.
(1.55 MB TIF)

Figure S3 QQ Plot.
(9.73 MB TIF)

Text S1 Supplementary Methods.
(0.13 MB DOC)
