# Supplementary material for: Identification of New Genetic Risk Variants for Type 2 Diabetes
Source: PLoS Genet. 2010 Sep 16;6(9):e1001127. doi: 10.1371/journal.pgen.1001127 (PMC2940731; doi:10.1371/journal.pgen.1001127)
Supplement: Table S1 — Association of 65 SNPs included in Replication Set I with T2D risk. (0.13 MB DOC) [file pgen.1001127.s001.doc]

**Table S1 Association of 65 SNPs included in Replication Set I with T2D risk**

|  |  |  | P value | | | |  |
| --- | --- | --- | --- | --- | --- | --- | --- |
| SNP | Region | Position | SBCS/SWHS | NHS/HPFS | KARE | SDCS/SP2 |  |
| rs17127361 | 1p31.3 | 65366870 | 0.002 | 0.013 | 0.767 |  |  |
| rs7536188 | 1p31.3 | 66860514 | 9.2 x 10^-4^ | 0.067 | 0.235 |  |  |
| rs11209138 | 1p31.3 | 67901227 | 3.0 x 10^-5^ | 0.063 | 0.715 |  |  |
| rs1408956 | 1p31.3 | 67909651 | 1.3 x 10^-4^ | 0.087 | 0.780 |  |  |
| rs6696952 | 1p22.3 | 87844551 | 0.002 | 0.082 | 0.320 |  |  |
| rs2815429 | 1p22.1 | 93363256 | 0.002 | 0.033 | 0.006 | 0.660 |  |
| rs3860336 | 1p21.3 | 95008870 | 5.3 x 10^-5^ | 0.015 | 0.653 |  |  |
| rs6541368 | 1p21.3 | 95010244 | 3.7 x 10^-5^ | 0.015 | 0.704 |  |  |
| rs832147 | 1q32.1 | 199536310 | 0.002 | 0.057 | 0.182 |  |  |
| rs1713223 | 2p24.1 | 21124775 | 3.6 x 10^-5^ | 0.159 | 0.561 |  |  |
| rs7575840 | 2p24.1 | 21126995 | 4.4 x 10^-5^ | 0.113 | 0.652 |  |  |
| rs1979121 | 2q33.1 | 200150849 | 0.002 | 0.013 | 0.173 |  |  |
| rs6728457 | 2q33.1 | 200154572 | 0.003 | 0.013 | 0.188 |  |  |
| rs6751662 | 2q34 | 212741363 | 1.6 x 10^-6^ | 0.041 | 0.701 |  |  |
| rs6793053 | 3p14.2 | 61919617 | 0.002 | 0.113 | 0.708 |  |  |
| rs1384180 | 3p13 | 73451339 | 0.002 | 0.055 | 0.136 |  |  |
| rs12637456 | 3q21.1 | 125227353 | 0.002 | 0.044 | 0.873 |  |  |
| rs1320186 | 3q21.1 | 125286944 | 0.002 | 0.035 | 0.456 |  |  |
| rs17602500 | 3q24 | 145148707 | 4.6 x 10^-5^ | 0.343 | 0.989 |  |  |
| rs210773 | 4p15.2 | 27037551 | 0.004 | 0.021 | 0.685 |  |  |
| rs2306601 | 4q12 | 53434993 | 0.004 | 0.075 | 0.786 |  |  |
| rs10018310 | 4q22.1 | 90806714 | 5.4 x 10^-4^ | 0.085 | 0.449 |  |  |
| rs10053085 | 5p15.31 | 6895007 | 0.004 | 0.181 | 0.861 |  |  |
| rs1010966 | 5q32 | 146635674 | 2.0 x 10^-4^ | 0.140 | 0.725 |  |  |
| rs9313375 | 5q34 | 166742728 | 0.003 | 0.125 | 0.596 |  |  |
| rs7452753 | 6q16.3 | 101160447 | 0.001 | 0.013 | 0.302 |  |  |
| rs9373571 | 6q16.3 | 101240555 | 0.001 | 0.024 | 0.175 |  |  |
| rs17504676 | 7q21.11 | 82970533 | 0.005 | 0.063 | 0.674 |  |  |
| rs209973 | 8p22 | 17595134 | 0.005 | 0.024 | 0.227 |  |  |
| rs1563325 | 8q12.3 | 63965379 | 0.004 | 0.168 | 0.143 |  |  |
| rs10755927 | 8q24.23 | 126698776 | 0.005 | 0.150 | 0.483 |  |  |
| rs1935341 | 9q21.13 | 73317823 | 2.7 x 10^-4^ | 0.222 | 0.916 |  |  |
| rs11142852 | 9q21.13 | 73307385 | 3.4 x 10^-4^ | 0.374 | 0.850 |  |  |
| rs7851025 | 9q22.33 | 98980793 | 1.3 x 10^-4^ | 0.013 | 0.832 |  |  |
| rs7915509 | 10p15.3 | 2276078 | 0.002 | 0.063 | 0.747 |  |  |
| rs1041598 | 10p15.3 | 2280492 | 0.002 | 0.105 | 0.477 |  |  |
| rs10906115 | 10p13 | 12355003 | 0.014 | 0.023 | 0.022 | 0.756 |  |
| rs11003226 | 10q21.1 | 54294628 | 7.5 x 10^-4^ | 0.087 | 0.727 |  |  |
| rs10749466 | 10q26.13 | 124139342 | 9.8 x 10^-4^ | 0.027 | 0.766 |  |  |
| rs10751301 | 11q14.1 | 78372286 | 0.005 | 1.0 x 10^-4^ | 0.111 | 0.392 |  |
| rs11237675 | 11q14.1 | 78375191 | 0.004 | 2.5 x 10^-4^ | 0.345 | 0.210 |  |
| rs2373547 | 11q14.1 | 80007427 | 6.6 x 10^-4^ | 0.141 | 0.925 |  |  |
| rs6589964 | 11q24.1 | 122375893 | 0.002 | 0.132 | 0.559 |  |  |
| rs10790965 | 11q24.3 | 127998557 | 8.1 x 10^-4^ | 0.036 | 0.439 |  |  |
| rs2099984 | 12q13.11 | 44823390 | 0.003 | 0.228 | 0.580 |  |  |
| rs10778857 | 12q21.31 | 80983297 | 8.4 x 10^-4^ | 0.141 | 0.388 |  |  |
| rs1120107 | 12q22 | 94311663 | 0.001 | 0.061 | 0.879 |  |  |
| rs10507349 | 13q12.13 | 25679528 | 0.005 | 6.6 x 10^-4^ | 0.287 | 0.756 |  |
| rs7139998 | 13q13.3 | 37174391 | 0.004 | 0.044 | 0.762 |  |  |
| rs1937076 | 13q21.33 | 70303653 | 0.003 | 0.100 | 0.566 |  |  |
| rs1215468 | 13q31.1 | 79605430 | 0.001 | 0.144 | 0.074 |  |  |
| rs1359790 | 13q31.1 | 79615157 | 4.4 x 10^-4^ | 0.109 | 0.111 | 0.174 |  |
| rs8029466 | 14q13.1 | 26761581 | 7.8 x 10^-4^ | 0.005 | 0.354 |  |  |
| rs8030240 | 15q22.2 | 60186856 | 0.002 | 0.004 | 0.011 |  |  |
| rs1436955 | 15q22.2 | 60191674 | 0.009 | 0.001 | 0.014 | 0.554 |  |
| rs16941593 | 15q25.3 | 86621433 | 1.5 x 10^-4^ | 0.090 | 0.103 |  |  |
| rs11073418 | 15q26.2 | 93448799 | 1.3 x 10^-4^ | 0.140 | 0.055 | 0.277 |  |
| rs2298613 | 18q12.1 | 27311717 | 0.004 | 0.040 | 0.293 |  |  |
| rs9304099 | 18q12.1 | 27340854 | 0.004 | 0.006 |  |  |  |
| rs12457182 | 18q21.1 | 45429214 | 2.7 x 10^-4^ | 0.037 | 0.945 |  |  |
| rs322520 | 18q21.2 | 47435180 | 0.004 | 0.055 | 0.720 |  |  |
| rs2281315 | 20q13.2 | 49304431 | 6.1 x 10^-4^ | 0.015 | 0.550 |  |  |
| rs6123004 | 20q13.2 | 49311277 | 0.002 | 0.031 | 0.979 |  |  |
| rs2179689 | 20q13.2 | 49313621 | 0.002 | 0.026 | 0.993 |  |  |
| rs6611276 | 23p11.3 | 46357610 | 7.4 x 10^-4^ | 0.113 |  |  |  |
