# Supplementary material for: Identification of New Genetic Risk Variants for Type 2 Diabetes
Source: PLoS Genet. 2010 Sep 16;6(9):e1001127. doi: 10.1371/journal.pgen.1001127 (PMC2940731; doi:10.1371/journal.pgen.1001127)
Supplement: Table S2 — Association of the top 4 SNPs with T2D risk with additional adjustments. (0.07 MB DOC) [file pgen.1001127.s002.doc]

Table S2 Association of the top 4 SNPs with T2D risk with additional adjustments

|  |  | Number | | Frequency | | OR (95% CI)^b^ | | |  |
| --- | --- | --- | --- | --- | --- | --- | --- | --- | --- |
| SNP | Study Set | Cases | Controls | Cases | Controls | Heterozygous | Homozygous | Per allele | P |
| rs10906115 | GWA scan | 1019 | 1710 | 0.65 | 0.62 | 1.23 (0.93- 1.62) | 1.45 (1.10- 1.92) | 1.20 (1.05- 1.36) | 0.006 |
|  | Replication Set I | 2562 | 3031 | 0.61 | 0.59 | 1.14 (0.96- 1.35) | 1.24 (1.04- 1.47) | 1.11 (1.02- 1.20) | 0.016 |
|  | Replication Set II^a^ | 2010 | 1945 | 0.62 | 0.61 | 1.06 (0.81- 1.37) | 1.07 (0.82- 1.40) | 1.03 (0.91- 1.17) | 0.65 |
|  | Replication Set III | 3115 | 4944 | 0.65 | 0.62 | 1.24 (1.06- 1.44) | 1.40 (1.20- 1.64) | 1.17 (1.09- 1.26) | 1.9 x 10^-5^ |
|  | Combined^a^ | 8706 | 11630 | 0.63 | 0.61 | 1.18 (1.07- 1.30) | 1.30 (1.18- 1.43) | 1.13 (1.08- 1.18) | 1.3 x 10^-7^ |
|  |  |  |  |  |  |  |  |  |  |
| rs1359790 | GWA scan | 1009 | 1690 | 0.75 | 0.71 | 1.33 (0.94- 1.90) | 1.56 (1.11- 2.20) | 1.21 (1.06- 1.39) | 0.006 |
|  | Replication Set I | 2571 | 3036 | 0.74 | 0.73 | 1.17 (0.92- 1.48) | 1.24 (0.99- 1.57) | 1.09 (0.99- 1.19) | 0.069 |
|  | Replication Set II^a^ | 2010 | 1945 | 0.72 | 0.71 | 1.10 (0.79- 1.56) | 1.20 (0.86- 1.66) | 1.09 (0.95- 1.25) | 0.22 |
|  | Replication Set III | 3117 | 4907 | 0.74 | 0.71 | 1.24 (1.02- 1.51) | 1.47 (1.21- 1.78) | 1.20 (1.11- 1.30) | 5.3 x 10^-6^ |
|  | Combined^a^ | 8707 | 11578 | 0.74 | 0.71 | 1.20 (1.06- 1.36) | 1.36 (1.20- 1.53) | 1.15 (1.09- 1.21) | 3.1 x 10^-8^ |
|  |  |  |  |  |  |  |  |  |  |
| rs1436955 | GWA scan | 1019 | 1709 | 0.79 | 0.75 | 0.99 (0.65- 1.50) | 1.28 (0.85- 1.93) | 1.22 (1.05- 1.42) | 0.008 |
|  | Replication Set I | 2590 | 3052 | 0.76 | 0.73 | 1.14 (0.90- 1.45) | 1.29 (1.02- 1.63) | 1.13 (1.03- 1.24) | 0.009 |
|  | Replication Set II^a^ | 2010 | 1945 | 0.81 | 0.80 | 0.98 (0.62- 1.57) | 1.09 (0.69- 1.71) | 1.08 (0.93- 1.26) | 0.33 |
|  | Replication Set III | 3126 | 4944 | 0.78 | 0.76 | 1.02 (0.81- 1.28) | 1.14 (0.92- 1.42) | 1.09 (1.01- 1.19) | 0.032 |
|  | Combined^a^ | 8745 | 11650 | 0.78 | 0.76 | 1.07 (0.92- 1.23) | 1.22 (1.06- 1.40) | 1.12 (1.07- 1.19) | 1.1 x 10^-5^ |
|  |  |  |  |  |  |  |  |  |  |
| rs10751301 | GWA scan | 1018 | 1710 | 0.24 | 0.21 | 1.17 (0.97- 1.40) | 1.69 (1.14- 2.49) | 1.23 (1.06- 1.42) | 0.006 |
|  | Replication Set I | 2590 | 3052 | 0.54 | 0.50 | 1.29 (1.12- 1.49) | 1.39 (1.18- 1.63) | 1.17 (1.08- 1.27) | 1.2 x 10^-4^ |
|  | Replication Set II^a^ | 2010 | 1945 | 0.22 | 0.21 | 1.19 (0.98- 1.43) | 0.97 (0.62- 1.51) | 1.10 (0.94- 1.28) | 0.22 |
|  | Replication Set III | 3123 | 4938 | 0.22 | 0.21 | 1.01 (0.91- 1.12) | 1.01 (0.80- 1.27) | 1.01 (0.93- 1.10) | 0.87 |
|  | Combined^a^ | 8741 | 11645 | 0.32 | 0.29 | 1.12 (1.05- 1.20) | 1.22 (1.09- 1.36) | 1.11 (1.06- 1.17) | 4.1 x 10^-5^ |

Notes:

a. No individual data was available for the Korean study, so it was not included in Replication Set II or the combined analyses.

b. Analyses were adjusted for age (continuous), BMI (continuous), gender (when possible), smoking, and study site (when possible).
