# Supplementary material for: Identification of New Genetic Risk Variants for Type 2 Diabetes
Source: PLoS Genet. 2010 Sep 16;6(9):e1001127. doi: 10.1371/journal.pgen.1001127 (PMC2940731; doi:10.1371/journal.pgen.1001127)
Supplement: Table S3 — Association of SNPs that are in LD with established SNPs in European-ancestry populations (for SNPs discovered in European ancestry populations) or with the reported SNPs in Asians (for SNPs discovered in Asians). (0.48 MB DOC) [file pgen.1001127.s003.doc]

Table S3 Association of SNPs that are in LD with established SNPs in European-ancestry populations (for SNPs discovered in European ancestry populations) or with the reported SNPs in Asians (for SNPs discovered in Asians)

| SNP Reported | SNP Tested | CHR | Position | r2 | Effect Allele | Effect Allele Frequency | Odds Ratio | P | Genotyping |
| --- | --- | --- | --- | --- | --- | --- | --- | --- | --- |
| rs10923931 | rs2793823 | 1 | 120239241 | 0.8 | A | 0.09 | 0.80 | 0.046 | typed |
| rs10923931 | rs2641348 | 1 | 120239407 | 0.925 | A | 0.97 | 1.06 | 0.76 | imputed |
| rs10923931 | rs2027447 | 1 | 120241552 | 0.925 | C | 0.03 | 0.94 | 0.77 | imputed |
| rs10923931 | rs2487568 | 1 | 120243521 | 0.8 | A | 0.88 | 1.27 | 0.04 | imputed |
| rs10923931 | rs2641346 | 1 | 120245063 | 0.925 | C | 0.97 | 1.06 | 0.78 | imputed |
| rs10923931 | rs2641352 | 1 | 120247586 | 0.925 | C | 0.03 | 0.95 | 0.79 | imputed |
| rs10923931 | rs3897478 | 1 | 120252713 | 0.925 | C | 0.03 | 0.96 | 0.81 | imputed |
| rs10923931 | rs947273 | 1 | 120252875 | 0.8 | A | 0.09 | 0.81 | 0.064 | imputed |
| rs10923931 | rs699779 | 1 | 120256833 | 0.952 | A | 0.97 | 1.04 | 0.83 | imputed |
| rs10923931 | rs835576 | 1 | 120257109 | 0.925 | C | 0.05 | 0.85 | 0.34 | imputed |
| rs10923931 | rs835575 | 1 | 120258086 | 0.925 | T | 0.03 | 0.96 | 0.84 | typed |
| rs10923931 | rs2793829 | 1 | 120262776 | 1 | C | 0.97 | 1.04 | 0.84 | imputed |
| rs10923931 | rs835574 | 1 | 120264753 | 0.858 | C | 0.91 | 1.22 | 0.079 | imputed |
| rs10923931 | rs835573 | 1 | 120265688 | 0.858 | C | 0.91 | 1.22 | 0.08 | imputed |
| rs10923931 | rs2793830 | 1 | 120267631 | 0.858 | A | 0.10 | 0.82 | 0.069 | imputed |
| rs10923931 | rs2493394 | 1 | 120272747 | 1 | A | 0.97 | 1.04 | 0.84 | imputed |
| rs10923931 | rs2493392 | 1 | 120273380 | 1 | C | 0.97 | 1.04 | 0.84 | imputed |
| rs10923931 | rs2493391 | 1 | 120274030 | 1 | A | 0.03 | 0.96 | 0.84 | imputed |
| rs10923931 | rs2493389 | 1 | 120275701 | 1 | C | 0.97 | 1.04 | 0.84 | imputed |
| rs10923931 | rs6688004 | 1 | 120276030 | 0.858 | G | 0.09 | 0.82 | 0.083 | imputed |
| rs10923931 | rs2453056 | 1 | 120279521 | 0.858 | A | 0.09 | 0.82 | 0.083 | imputed |
| rs10923931 | rs5025718 | 1 | 120279808 | 0.858 | C | 0.91 | 1.21 | 0.083 | imputed |
| rs10923931 | rs2453058 | 1 | 120282232 | 1 | C | 0.97 | 1.04 | 0.84 | imputed |
| rs10923931 | rs2493420 | 1 | 120285944 | 0.858 | A | 0.09 | 0.82 | 0.083 | imputed |
| rs10923931 | rs2934387 | 1 | 120297029 | 1 | C | 0.03 | 0.96 | 0.84 | imputed |
| rs10923931 | rs7534585 | 1 | 120297642 | 1 | A | 0.97 | 1.04 | 0.84 | imputed |
| rs10923931 | rs7534586 | 1 | 120297650 | 1 | A | 0.97 | 1.04 | 0.84 | imputed |
| rs10923931 | rs4659250 | 1 | 120299609 | 0.858 | C | 0.91 | 1.21 | 0.084 | imputed |
| rs10923931 | rs10127888 | 1 | 120299773 | 1 | G | 0.03 | 0.96 | 0.84 | typed |
| rs10923931 | rs1493696 | 1 | 120307055 | 0.858 | A | 0.09 | 0.82 | 0.084 | imputed |
| rs10923931 | rs2934381 | 1 | 120307631 | 1 | A | 0.03 | 0.96 | 0.84 | typed |
| rs10923931 | rs2453044 | 1 | 120310047 | 0.858 | G | 0.09 | 0.82 | 0.072 | typed |
| rs10923931 | rs2493410 | 1 | 120310326 | 0.858 | C | 0.09 | 0.84 | 0.12 | imputed |
| rs10923931 | rs1493695 | 1 | 120314640 | 0.858 | A | 0.91 | 1.21 | 0.097 | imputed |
| rs10923931 | rs7515431 | 1 | 120315071 | 0.92 | C | 0.97 | 0.97 | 0.86 | imputed |
| rs10923931 | rs2641317 | 1 | 120318141 | 1 | C | 0.97 | 0.97 | 0.87 | imputed |
| rs10923931 | rs10494235 | 1 | 120322238 | 0.858 | A | 0.08 | 0.78 | 0.033 | typed |
| rs10923931 | rs2793831 | 1 | 120325425 | 1 | C | 0.03 | 0.99 | 0.95 | imputed |
| rs10923931 | rs1493694 | 1 | 120328505 | 1 | C | 0.97 | 1.01 | 0.96 | imputed |
| rs10923931 | rs2453040 | 1 | 120331273 | 1 | A | 0.97 | 0.99 | 0.97 | imputed |
| rs10923931 | rs327196 | 1 | 120370133 | 0.858 | A | 0.91 | 1.29 | 0.08 | imputed |
| rs7593730 | rs2966379 | 2 | 160831497 | 0.803 | C | 0.17 | 0.99 | 0.86 | imputed |
| rs7593730 | rs12692592 | 2 | 160871627 | 1 | G | 0.16 | 1.05 | 0.57 | typed |
| rs7593730 | rs4077463 | 2 | 160874480 | 1 | A | 0.16 | 0.97 | 0.71 | typed |
| rs7593730 | rs10929978 | 2 | 160878202 | 1 | C | 0.83 | 1.01 | 0.94 | imputed |
| rs7593730 | rs4589705 | 2 | 160884382 | 1 | T | 0.17 | 0.94 | 0.46 | typed |
| rs7593730 | rs12622664 | 2 | 160887602 | 0.95 | C | 0.17 | 0.97 | 0.74 | imputed |
| rs7593730 | rs12465609 | 2 | 160887731 | 1 | C | 0.83 | 1.02 | 0.78 | imputed |
| rs7593730 | rs4410242 | 2 | 160900316 | 1 | A | 0.17 | 0.98 | 0.80 | imputed |
| rs7593730 | rs10209870 | 2 | 160900791 | 1 | C | 0.83 | 1.02 | 0.83 | imputed |
| rs7593730 | rs12476804 | 2 | 160902501 | 0.95 | C | 0.17 | 0.99 | 0.95 | imputed |
| rs7593730 | rs1110209 | 2 | 160907744 | 1 | C | 0.83 | 1.01 | 0.95 | imputed |
| rs7593730 | rs10168504 | 2 | 160910834 | 0.95 | A | 0.83 | 1.00 | 0.98 | imputed |
| rs7593730 | rs9287795 | 2 | 160918034 | 0.95 | C | 0.16 | 1.02 | 0.80 | typed |
| rs7593730 | rs6724146 | 2 | 160918901 | 1 | C | 0.83 | 0.99 | 0.94 | imputed |
| rs7593730 | rs6718526 | 2 | 160922421 | 0.898 | T | 0.14 | 0.94 | 0.52 | typed |
| rs7593730 | rs6730051 | 2 | 160924967 | 0.95 | C | 0.83 | 1.00 | 0.98 | imputed |
| rs7593730 | rs4277476 | 2 | 160927427 | 0.95 | A | 0.17 | 0.99 | 0.94 | imputed |
| rs7593730 | rs11693602 | 2 | 160932904 | 0.95 | C | 0.16 | 0.96 | 0.63 | typed |
| rs7593730 | rs10929981 | 2 | 160942041 | 0.848 | C | 0.14 | 0.93 | 0.45 | imputed |
| rs7593730 | rs10929982 | 2 | 160944523 | 0.848 | C | 0.14 | 0.92 | 0.34 | typed |
| rs2943641 | rs2943653 | 2 | 226756015 | 0.802 | C | 0.10 | 0.87 | 0.24 | imputed |
| rs2943641 | rs952227 | 2 | 226770324 | 0.802 | A | 0.09 | 0.86 | 0.18 | imputed |
| rs2943641 | rs2943634 | 2 | 226776324 | 0.802 | A | 0.09 | 0.86 | 0.18 | typed |
| rs2943641 | rs1399627 | 2 | 226791655 | 1 | A | 0.06 | 0.84 | 0.15 | imputed |
| rs2943641 | rs1515104 | 2 | 226800649 | 0.982 | T | 0.07 | 0.88 | 0.29 | typed |
| rs2943641 | rs2176040 | 2 | 226801046 | 1 | A | 0.06 | 0.83 | 0.15 | imputed |
| rs2943641 | rs2943640 | 2 | 226801829 | 0.966 | A | 0.07 | 0.85 | 0.19 | typed |
| rs2943641 | rs2943645 | 2 | 226807424 | 0.966 | C | 0.06 | 0.82 | 0.12 | imputed |
| rs2943641 | rs2943646 | 2 | 226807778 | 0.966 | A | 0.07 | 0.84 | 0.16 | typed |
| rs2943641 | rs2972147 | 2 | 226808098 | 0.966 | T | 0.07 | 0.83 | 0.14 | typed |
| rs2943641 | rs2972146 | 2 | 226808942 | 0.966 | G | 0.06 | 0.81 | 0.10 | imputed |
| rs2943641 | rs2972144 | 2 | 226809655 | 1 | A | 0.07 | 0.81 | 0.099 | typed |
| rs2943641 | rs2138157 | 2 | 226811961 | 0.898 | A | 0.07 | 0.82 | 0.12 | typed |
| rs2943641 | rs2943650 | 2 | 226814165 | 1 | C | 0.07 | 0.82 | 0.12 | imputed |
| rs2943641 | rs2972136 | 2 | 226815279 | 1 | A | 0.07 | 0.85 | 0.18 | typed |
| rs2943641 | rs2943652 | 2 | 226816690 | 0.966 | C | 0.07 | 0.82 | 0.12 | imputed |
| rs2943641 | rs2972143 | 2 | 226824609 | 1 | A | 0.07 | 0.82 | 0.12 | imputed |
| rs2943641 | rs2943656 | 2 | 226830162 | 0.898 | A | 0.08 | 0.85 | 0.17 | typed |
| rs2943641 | rs1515110 | 2 | 226830460 | 0.898 | G | 0.08 | 0.85 | 0.15 | imputed |
| rs2943641 | rs1515108 | 2 | 226831330 | 0.898 | C | 0.08 | 0.83 | 0.12 | typed |
| rs2943641 | rs2943657 | 2 | 226831683 | 0.898 | C | 0.08 | 0.81 | 0.069 | typed |
| rs2943641 | rs2943658 | 2 | 226831828 | 0.898 | A | 0.08 | 0.85 | 0.15 | imputed |
| rs2943641 | rs2943659 | 2 | 226833498 | 0.898 | A | 0.92 | 1.18 | 0.15 | imputed |
| rs2943641 | rs1399626 | 2 | 226833660 | 0.898 | T | 0.08 | 0.87 | 0.21 | typed |
| rs2943641 | rs2943660 | 2 | 226836787 | 0.898 | G | 0.92 | 1.18 | 0.17 | imputed |
| rs2943641 | rs1515099 | 2 | 226837143 | 0.898 | G | 0.08 | 0.86 | 0.20 | typed |
| rs2943641 | rs1515100 | 2 | 226837161 | 0.898 | C | 0.08 | 0.84 | 0.14 | typed |
| rs2943641 | rs2713539 | 2 | 226845369 | 0.898 | C | 0.93 | 1.17 | 0.17 | imputed |
| rs2943641 | rs2713540 | 2 | 226848730 | 0.898 | A | 0.07 | 0.85 | 0.17 | imputed |
| rs2943641 | rs2673131 | 2 | 226849492 | 0.865 | A | 0.07 | 0.85 | 0.18 | imputed |
| rs2943641 | rs2713541 | 2 | 226850012 | 0.898 | A | 0.08 | 0.86 | 0.21 | typed |
| rs2943641 | rs2673135 | 2 | 226859840 | 0.898 | A | 0.93 | 1.19 | 0.14 | imputed |
| rs2943641 | rs2673142 | 2 | 226864889 | 0.898 | G | 0.10 | 0.94 | 0.55 | typed |
| rs2943641 | rs2713548 | 2 | 226864906 | 0.898 | C | 0.07 | 0.84 | 0.13 | imputed |
| rs2943641 | rs2458993 | 2 | 226864974 | 0.865 | C | 0.07 | 0.84 | 0.13 | imputed |
| rs2943641 | rs2713549 | 2 | 226865453 | 0.898 | A | 0.07 | 0.84 | 0.13 | imputed |
| rs2943641 | rs2673141 | 2 | 226867421 | 0.898 | A | 0.93 | 1.20 | 0.12 | imputed |
| rs2943641 | rs2673140 | 2 | 226868111 | 0.898 | G | 0.08 | 0.84 | 0.13 | typed |
| rs2943641 | rs2713559 | 2 | 226872748 | 0.898 | C | 0.07 | 0.83 | 0.12 | imputed |
| rs2943641 | rs2713557 | 2 | 226879847 | 0.898 | C | 0.93 | 1.20 | 0.11 | imputed |
| rs2943641 | rs1515116 | 2 | 226884337 | 0.843 | G | 0.08 | 0.81 | 0.071 | typed |
| rs2943641 | rs925735 | 2 | 226887874 | 0.898 | C | 0.08 | 0.83 | 0.12 | imputed |
| rs2943641 | rs908252 | 2 | 226888863 | 0.898 | G | 0.08 | 0.86 | 0.20 | imputed |
| rs2943641 | rs2713552 | 2 | 226889927 | 0.865 | G | 0.08 | 0.87 | 0.24 | imputed |
| rs1801282 | rs11709077 | 3 | 12311507 | 1 | A | 0.05 | 0.96 | 0.75 | typed |
| rs1801282 | rs13083375 | 3 | 12340308 | 1 | G | 0.94 | 1.04 | 0.78 | imputed |
| rs1801282 | rs13064760 | 3 | 12344401 | 1 | C | 0.94 | 1.04 | 0.79 | imputed |
| rs1801282 | rs4684847 | 3 | 12361337 | 1 | C | 0.94 | 1.03 | 0.84 | imputed |
| rs1801282 | rs17036326 | 3 | 12364313 | 1 | G | 0.06 | 1.10 | 0.51 | typed |
| rs1801282 | rs17036328 | 3 | 12365484 | 1 | C | 0.06 | 0.97 | 0.85 | imputed |
| rs1801282 | rs6802898 | 3 | 12366207 | 1 | C | 0.94 | 1.03 | 0.85 | imputed |
| rs1801282 | rs2197423 | 3 | 12366583 | 1 | A | 0.06 | 0.98 | 0.86 | imputed |
| rs1801282 | rs7647481 | 3 | 12366813 | 1 | A | 0.06 | 0.98 | 0.87 | imputed |
| rs1801282 | rs7649970 | 3 | 12367272 | 1 | C | 0.94 | 1.02 | 0.87 | imputed |
| rs1801282 | rs1899951 | 3 | 12369840 | 1 | T | 0.06 | 1.08 | 0.58 | typed |
| rs1801282 | rs2881654 | 3 | 12371955 | 1 | A | 0.06 | 0.98 | 0.87 | imputed |
| rs4607103 | rs4616635 | 3 | 64677315 | 0.901 | G | 0.22 | 0.94 | 0.39 | typed |
| rs4607103 | rs4411878 | 3 | 64678705 | 0.948 | T | 0.19 | 0.94 | 0.44 | typed |
| rs4607103 | rs4324463 | 3 | 64683668 | 1 | C | 0.82 | 1.07 | 0.43 | imputed |
| rs4607103 | rs2371767 | 3 | 64693298 | 0.901 | C | 0.18 | 0.94 | 0.43 | imputed |
| rs4607103 | rs2194091 | 3 | 64700525 | 0.946 | G | 0.37 | 0.99 | 0.86 | typed |
| rs4607103 | rs7433808 | 3 | 64702126 | 1 | T | 0.18 | 0.90 | 0.21 | typed |
| rs4607103 | rs7638389 | 3 | 64704932 | 0.948 | A | 0.64 | 0.99 | 0.91 | imputed |
| rs4402960 | rs7648605 | 3 | 186973428 | 0.883 | G | 0.19 | 1.36 | 2.0 x 10^-4^ | imputed |
| rs4402960 | rs11706322 | 3 | 186979987 | 0.883 | T | 0.18 | 1.35 | 1.6 x 10^-4^ | typed |
| rs4402960 | rs4376068 | 3 | 186980329 | 1 | C | 0.25 | 1.30 | 2.8 x 10^-4^ | typed |
| rs4402960 | rs6801848 | 3 | 186981751 | 0.921 | C | 0.76 | 0.77 | 2.4 x 10^-4^ | imputed |
| rs4402960 | rs4481184 | 3 | 186988481 | 1 | C | 0.75 | 0.77 | 3.5 x 10^-4^ | imputed |
| rs4402960 | rs11716491 | 3 | 186988697 | 0.807 | C | 0.19 | 1.37 | 9.3 x 10^-5^ | imputed |
| rs4402960 | rs11705729 | 3 | 186989993 | 1 | A | 0.73 | 0.79 | 0.001 | imputed |
| rs4402960 | rs11929397 | 3 | 186992884 | 1 | C | 0.25 | 1.29 | 3.9 x 10^-4^ | imputed |
| rs4402960 | rs7633675 | 3 | 186993307 | 1 | G | 0.27 | 1.26 | 0.001 | imputed |
| rs4402960 | rs16860234 | 3 | 186993578 | 0.96 | A | 0.81 | 0.74 | 9.9 x 10^-5^ | imputed |
| rs4402960 | rs16860235 | 3 | 186995055 | 0.921 | A | 0.04 | 1.47 | 0.034 | imputed |
| rs4402960 | rs7640539 | 3 | 186995990 | 1 | A | 0.25 | 1.29 | 4.2 x 10^-4^ | imputed |
| rs4402960 | rs7651090 | 3 | 186996086 | 0.96 | G | 0.25 | 1.29 | 4.4 x 10^-4^ | typed |
| rs4402960 | rs6444081 | 3 | 186997087 | 1 | C | 0.25 | 1.29 | 4.1 x 10^-4^ | imputed |
| rs4402960 | rs7646518 | 3 | 186997625 | 1 | C | 0.25 | 1.29 | 4.0 x 10^-4^ | imputed |
| rs4402960 | rs7637773 | 3 | 186998329 | 0.961 | A | 0.25 | 1.29 | 4.0 x 10^-4^ | imputed |
| rs4402960 | rs4686696 | 3 | 186999214 | 1 | A | 0.25 | 1.29 | 3.9 x 10^-4^ | imputed |
| rs4402960 | rs6767484 | 3 | 187003272 | 1 | A | 0.75 | 0.78 | 3.9 x 10^-4^ | imputed |
| rs4402960 | rs6767577 | 3 | 187003426 | 0.883 | C | 0.18 | 1.38 | 5.3 x 10^-5^ | typed |
| rs4402960 | rs7640744 | 3 | 187005141 | 0.96 | A | 0.19 | 1.35 | 1.1 x 10^-4^ | typed |
| rs4402960 | rs13060777 | 3 | 187007893 | 0.883 | A | 0.82 | 0.73 | 5.3 x 10^-5^ | imputed |
| rs4402960 | rs11711477 | 3 | 187009384 | 1 | A | 0.27 | 1.29 | 3.4 x 10^-4^ | typed |
| rs4402960 | rs1470579 | 3 | 187011774 | 1 | C | 0.26 | 1.25 | 0.002 | typed |
| rs4402960 | rs1470580 | 3 | 187011868 | 1 | A | 0.27 | 1.26 | 0.001 | imputed |
| rs4402960 | rs1470581 | 3 | 187012021 | 0.883 | A | 0.81 | 0.75 | 2.0 x 10^-4^ | imputed |
| rs4402960 | rs6769511 | 3 | 187012984 | 1 | C | 0.27 | 1.26 | 0.001 | imputed |
| rs4402960 | rs9837654 | 3 | 187013016 | 0.883 | A | 0.20 | 1.33 | 2.2 x 10^-4^ | typed |
| rs4402960 | rs9859406 | 3 | 187017176 | 1 | A | 0.27 | 1.26 | 0.001 | imputed |
| rs10010131 | rs4688982 | 4 | 6317902 | 0.92 | C | 0.03 | 1.08 | 0.73 | imputed |
| rs10010131 | rs4689388 | 4 | 6320957 | 0.959 | A | 0.96 | 0.89 | 0.51 | imputed |
| rs10010131 | rs4320200 | 4 | 6321944 | 0.92 | A | 0.04 | 1.08 | 0.65 | imputed |
| rs10010131 | rs4689391 | 4 | 6331350 | 0.959 | A | 0.96 | 0.92 | 0.59 | imputed |
| rs10010131 | rs3889821 | 4 | 6333497 | 0.959 | C | 0.04 | 1.09 | 0.59 | imputed |
| rs10010131 | rs4688986 | 4 | 6336698 | 0.959 | A | 0.96 | 0.92 | 0.59 | imputed |
| rs10010131 | rs4689393 | 4 | 6338142 | 0.809 | C | 0.96 | 0.89 | 0.46 | imputed |
| rs10010131 | rs4688987 | 4 | 6338230 | 0.959 | C | 0.04 | 1.13 | 0.46 | imputed |
| rs10010131 | rs12508672 | 4 | 6338866 | 1 | A | 0.96 | 0.89 | 0.46 | imputed |
| rs10010131 | rs4458523 | 4 | 6340887 | 1 | T | 0.05 | 1.05 | 0.74 | typed |
| rs10010131 | rs4689394 | 4 | 6341904 | 1 | C | 0.04 | 1.14 | 0.41 | typed |
| rs10010131 | rs4293850 | 4 | 6342921 | 1 | A | 0.04 | 1.09 | 0.59 | typed |
| rs10010131 | rs11732178 | 4 | 6343544 | 0.959 | C | 0.96 | 0.91 | 0.56 | imputed |
| rs10010131 | rs5018647 | 4 | 6343700 | 1 | A | 0.04 | 1.14 | 0.39 | imputed |
| rs10010131 | rs5018648 | 4 | 6343719 | 1 | C | 0.04 | 1.15 | 0.39 | typed |
| rs10010131 | rs10012946 | 4 | 6344251 | 1 | T | 0.05 | 1.06 | 0.70 | typed |
| rs10010131 | rs13101355 | 4 | 6344347 | 0.917 | C | 0.95 | 0.95 | 0.71 | imputed |
| rs10010131 | rs4416547 | 4 | 6344868 | 1 | A | 0.95 | 0.95 | 0.71 | imputed |
| rs10010131 | rs4308429 | 4 | 6344902 | 1 | A | 0.05 | 1.06 | 0.71 | imputed |
| rs10010131 | rs4467645 | 4 | 6345206 | 1 | C | 0.05 | 1.06 | 0.71 | imputed |
| rs10010131 | rs13128674 | 4 | 6345418 | 1 | C | 0.05 | 1.06 | 0.71 | imputed |
| rs10010131 | rs13103357 | 4 | 6345445 | 1 | A | 0.05 | 1.01 | 0.96 | imputed |
| rs10010131 | rs13108780 | 4 | 6345531 | 1 | A | 0.06 | 1.10 | 0.52 | imputed |
| rs10010131 | rs12649341 | 4 | 6345660 | 1 | A | 0.95 | 0.95 | 0.71 | imputed |
| rs10010131 | rs10937717 | 4 | 6345988 | 1 | A | 0.95 | 0.95 | 0.71 | imputed |
| rs10010131 | rs10937718 | 4 | 6346118 | 1 | C | 0.95 | 0.95 | 0.71 | imputed |
| rs10010131 | rs10937719 | 4 | 6346223 | 0.835 | C | 0.05 | 1.06 | 0.71 | imputed |
| rs10010131 | rs10937720 | 4 | 6346352 | 1 | A | 0.05 | 1.06 | 0.71 | imputed |
| rs10010131 | rs6446480 | 4 | 6346466 | 1 | C | 0.05 | 1.06 | 0.71 | imputed |
| rs10010131 | rs6446482 | 4 | 6346594 | 0.958 | C | 0.05 | 1.05 | 0.73 | imputed |
| rs10010131 | rs6820509 | 4 | 6346651 | 0.816 | A | 0.14 | 1.10 | 0.45 | imputed |
| rs10010131 | rs13130845 | 4 | 6348547 | 0.959 | A | 0.05 | 1.06 | 0.71 | imputed |
| rs10010131 | rs4481292 | 4 | 6349276 | 0.92 | C | 0.05 | 1.06 | 0.71 | imputed |
| rs10010131 | rs4689397 | 4 | 6350288 | 0.883 | A | 0.95 | 0.95 | 0.71 | imputed |
| rs10010131 | rs3821943 | 4 | 6350841 | 0.883 | C | 0.15 | 1.12 | 0.38 | imputed |
| rs10010131 | rs1801206 | 4 | 6353608 | 0.92 | C | 0.05 | 1.05 | 0.73 | typed |
| rs10010131 | rs1801214 | 4 | 6353923 | 0.917 | C | 0.04 | 1.11 | 0.50 | imputed |
| rs10010131 | rs734312 | 4 | 6354255 | 0.92 | A | 0.85 | 0.98 | 0.81 | imputed |
| rs10010131 | rs1046314 | 4 | 6354856 | 0.883 | G | 0.05 | 1.04 | 0.79 | typed |
| rs10010131 | rs1046320 | 4 | 6355245 | 0.962 | G | 0.09 | 1.08 | 0.47 | typed |
| rs10010131 | rs3200 | 4 | 6355779 | 0.92 | C | 0.05 | 1.06 | 0.68 | imputed |
| rs10010131 | rs4580722 | 4 | 6357045 | 0.883 | A | 0.95 | 0.94 | 0.67 | imputed |
| rs10010131 | rs10937721 | 4 | 6357664 | 0.92 | C | 0.95 | 0.94 | 0.67 | imputed |
| rs10946398 | rs9295474 | 6 | 20760696 | 1 | C | 0.57 | 0.85 | 0.009 | imputed |
| rs10946398 | rs4712522 | 6 | 20764779 | 1 | C | 0.58 | 0.83 | 0.004 | imputed |
| rs10946398 | rs4712523 | 6 | 20765543 | 1 | A | 0.57 | 0.85 | 0.009 | imputed |
| rs10946398 | rs6906327 | 6 | 20767438 | 0.816 | A | 0.42 | 1.20 | 0.004 | imputed |
| rs10946398 | rs6456367 | 6 | 20767566 | 1 | A | 0.42 | 1.20 | 0.004 | imputed |
| rs10946398 | rs6456368 | 6 | 20767785 | 1 | C | 0.42 | 1.19 | 0.009 | typed |
| rs10946398 | rs6456369 | 6 | 20768344 | 0.846 | C | 0.43 | 1.21 | 0.004 | typed |
| rs10946398 | rs7774594 | 6 | 20769122 | 1 | A | 0.42 | 1.20 | 0.005 | imputed |
| rs10946398 | rs7754840 | 6 | 20769229 | 1 | C | 0.43 | 1.20 | 0.005 | typed |
| rs10946398 | rs9460544 | 6 | 20769508 | 1 | G | 0.58 | 0.83 | 0.005 | imputed |
| rs10946398 | rs9460545 | 6 | 20769529 | 1 | C | 0.42 | 1.20 | 0.005 | imputed |
| rs10946398 | rs4712525 | 6 | 20770945 | 1 | C | 0.58 | 0.83 | 0.005 | imputed |
| rs10946398 | rs4712526 | 6 | 20771014 | 1 | A | 0.42 | 1.20 | 0.005 | imputed |
| rs10946398 | rs9460546 | 6 | 20771611 | 1 | G | 0.42 | 1.22 | 0.002 | typed |
| rs10946398 | rs7748382 | 6 | 20773528 | 1 | A | 0.42 | 1.20 | 0.005 | imputed |
| rs10946398 | rs7772603 | 6 | 20773925 | 1 | C | 0.42 | 1.20 | 0.004 | imputed |
| rs10946398 | rs7752780 | 6 | 20774001 | 1 | A | 0.42 | 1.21 | 0.004 | imputed |
| rs10946398 | rs7752906 | 6 | 20774034 | 1 | A | 0.42 | 1.21 | 0.004 | imputed |
| rs10946398 | rs9358356 | 6 | 20775361 | 1 | C | 0.42 | 1.21 | 0.003 | imputed |
| rs864745 | rs10274928 | 7 | 28108613 | 0.855 | G | 0.01 | 1.56 | 0.15 | typed |
| rs864745 | rs1635852 | 7 | 28155936 | 0.967 | C | 0.24 | 1.03 | 0.73 | imputed |
| rs864745 | rs849133 | 7 | 28158805 | 0.967 | C | 0.76 | 0.98 | 0.73 | imputed |
| rs864745 | rs849134 | 7 | 28162747 | 0.967 | A | 0.76 | 0.98 | 0.73 | imputed |
| rs13266634 | rs3802177 | 8 | 118254206 | 1 | A | 0.43 | 0.85 | 0.011 | imputed |
| rs13266634 | rs11558471 | 8 | 118254914 | 0.957 | A | 0.57 | 1.18 | 0.012 | imputed |
| rs13266634 | rs11774700 | 8 | 118289451 | 0.828 | C | 0.43 | 0.86 | 0.017 | imputed |
| rs564398 | rs1063192 | 9 | 21993367 | 0.9 | A | 0.81 | 1.04 | 0.70 | imputed |
| rs564398 | rs2069418 | 9 | 21999698 | 0.812 | C | 0.89 | 1.07 | 0.51 | imputed |
| rs564398 | rs573687 | 9 | 22001642 | 0.83 | A | 0.11 | 0.93 | 0.51 | imputed |
| rs564398 | rs523096 | 9 | 22009129 | 0.865 | G | 0.12 | 0.93 | 0.49 | typed |
| rs564398 | rs518394 | 9 | 22009673 | 0.884 | C | 0.12 | 0.94 | 0.54 | typed |
| rs564398 | rs615552 | 9 | 22016077 | 0.897 | C | 0.12 | 0.94 | 0.54 | imputed |
| rs564398 | rs543830 | 9 | 22016639 | 0.965 | A | 0.88 | 1.06 | 0.54 | imputed |
| rs564398 | rs679038 | 9 | 22019080 | 0.965 | A | 0.12 | 0.94 | 0.52 | imputed |
| rs564398 | rs7865618 | 9 | 22021005 | 0.932 | A | 0.88 | 1.07 | 0.52 | imputed |
| rs564398 | rs634537 | 9 | 22022152 | 0.965 | G | 0.12 | 0.94 | 0.53 | imputed |
| rs564398 | rs2157719 | 9 | 22023366 | 0.897 | C | 0.12 | 0.94 | 0.53 | imputed |
| rs564398 | rs1008878 | 9 | 22026112 | 0.863 | G | 0.12 | 0.94 | 0.53 | imputed |
| rs564398 | rs1556515 | 9 | 22026367 | 0.83 | C | 0.12 | 0.94 | 0.53 | imputed |
| rs564398 | rs1333037 | 9 | 22030765 | 0.863 | C | 0.12 | 0.95 | 0.60 | imputed |
| rs564398 | rs1412829 | 9 | 22033926 | 0.965 | A | 0.88 | 1.05 | 0.61 | imputed |
| rs564398 | rs1360589 | 9 | 22035317 | 0.83 | C | 0.12 | 0.97 | 0.72 | imputed |
| rs564398 | rs944801 | 9 | 22041670 | 0.83 | C | 0.88 | 1.04 | 0.72 | imputed |
| rs564398 | rs7030641 | 9 | 22044040 | 0.83 | C | 0.13 | 1.01 | 0.93 | imputed |
| rs10811661 | rs2383208 | 9 | 22122076 | 1 | A | 0.56 | 1.24 | 8.7 x 10^-4^ | imputed |
| rs10811661 | rs10965250 | 9 | 22123284 | 0.95 | A | 0.46 | 0.80 | 4.6 x 10^-4^ | imputed |
| rs12779790 | rs11257622 | 10 | 12335345 | 0.837 | C | 0.33 | 1.00 | 0.95 | imputed |
| rs12779790 | rs4747969 | 10 | 12337236 | 0.837 | C | 0.33 | 1.00 | 0.95 | typed |
| rs12779790 | rs11257655 | 10 | 12347900 | 0.802 | C | 0.47 | 0.81 | 0.004 | imputed |
| rs5015480 | rs1111875 | 10 | 94452862 | 1 | C | 0.28 | 1.27 | 7.6 x 10^-4^ | imputed |
| rs5015480 | rs12778642 | 10 | 94454287 | 0.904 | G | 0.22 | 1.22 | 0.011 | imputed |
| rs5015480 | rs10882102 | 10 | 94456475 | 1 | C | 0.82 | 0.70 | 1.0 x 10^-5^ | imputed |
| rs7901695 | rs4506565 | 10 | 114746031 | 0.878 | T | 0.04 | 1.43 | 0.025 | typed |
| rs7901695 | rs7903146 | 10 | 114748339 | 0.8 | C | 0.97 | 0.70 | 0.029 | imputed |
| rs2237892 | rs7480855 | 11 | 2792661 | 0.839 | G | 0.37 | 0.85 | 0.013 | typed |
| rs2237892 | rs2283228 | 11 | 2806106 | 0.864 | C | 0.36 | 0.82 | 0.003 | typed |
| rs2237892 | rs234852 | 11 | 2807358 | 0.804 | T | 0.37 | 0.85 | 0.011 | typed |
| rs5215 | rs7928810 | 11 | 17329019 | 0.902 | C | 0.37 | 1.18 | 0.010 | typed |
| rs5215 | rs2051772 | 11 | 17346426 | 0.902 | A | 0.62 | 0.84 | 0.007 | imputed |
| rs5215 | rs10832778 | 11 | 17350649 | 0.902 | C | 0.38 | 1.19 | 0.007 | imputed |
| rs5215 | rs1557765 | 11 | 17360215 | 0.902 | C | 0.60 | 0.84 | 0.006 | imputed |
| rs5215 | rs1002226 | 11 | 17362193 | 0.948 | C | 0.40 | 1.19 | 0.005 | typed |
| rs5215 | rs2074314 | 11 | 17368397 | 0.932 | C | 0.39 | 1.20 | 0.004 | imputed |
| rs5215 | rs757110 | 11 | 17375053 | 0.899 | A | 0.59 | 0.83 | 0.005 | imputed |
| rs1495377 | rs1796336 | 12 | 69826038 | 0.842 | C | 0.35 | 1.04 | 0.58 | imputed |
| rs1495377 | rs1796337 | 12 | 69826353 | 0.842 | C | 0.65 | 0.96 | 0.55 | imputed |
| rs1495377 | rs1798086 | 12 | 69833786 | 1 | C | 0.73 | 1.02 | 0.74 | imputed |
| rs1495377 | rs1705237 | 12 | 69833997 | 0.967 | A | 0.26 | 0.98 | 0.78 | typed |
| rs1495377 | rs1614565 | 12 | 69835363 | 1 | A | 0.73 | 1.03 | 0.73 | imputed |
| rs1495377 | rs1798085 | 12 | 69836964 | 1 | C | 0.73 | 1.03 | 0.73 | imputed |
| rs1495377 | rs1798083 | 12 | 69849803 | 1 | C | 0.27 | 0.97 | 0.64 | imputed |
| rs1495377 | rs1510938 | 12 | 69854615 | 1 | G | 0.73 | 1.04 | 0.61 | imputed |
| rs1495377 | rs1611977 | 12 | 69856062 | 1 | G | 0.73 | 1.04 | 0.61 | imputed |
| rs1495377 | rs1603232 | 12 | 69858101 | 1 | A | 0.27 | 0.96 | 0.61 | imputed |
| rs1495377 | rs1603231 | 12 | 69861523 | 1 | A | 0.73 | 1.04 | 0.60 | imputed |
| rs1495377 | rs1495376 | 12 | 69863849 | 1 | C | 0.73 | 1.06 | 0.46 | imputed |
| rs7961581 | rs1353361 | 12 | 69894466 | 0.955 | A | 0.72 | 1.05 | 0.52 | imputed |
| rs7961581 | rs1353362 | 12 | 69899543 | 0.955 | C | 0.24 | 0.97 | 0.67 | imputed |
| rs7961581 | rs7306184 | 12 | 69904470 | 0.909 | C | 0.29 | 0.97 | 0.69 | imputed |
| rs7961581 | rs4760915 | 12 | 69920379 | 0.909 | C | 0.75 | 1.03 | 0.70 | imputed |
| rs7961581 | rs4760790 | 12 | 69921061 | 0.909 | A | 0.25 | 0.97 | 0.67 | imputed |
| rs7961581 | rs6581998 | 12 | 69942990 | 0.955 | C | 0.20 | 0.96 | 0.65 | imputed |
| rs8050136 | rs9937053 | 16 | 52357008 | 0.842 | A | 0.17 | 0.98 | 0.84 | imputed |
| rs8050136 | rs9928094 | 16 | 52357406 | 0.842 | A | 0.83 | 1.02 | 0.83 | imputed |
| rs8050136 | rs9939973 | 16 | 52358069 | 0.842 | A | 0.17 | 0.98 | 0.84 | typed |
| rs8050136 | rs9940646 | 16 | 52358130 | 0.813 | C | 0.83 | 1.02 | 0.83 | imputed |
| rs8050136 | rs9940128 | 16 | 52358255 | 0.842 | A | 0.17 | 0.99 | 0.87 | typed |
| rs8050136 | rs1421085 | 16 | 52358455 | 0.934 | C | 0.12 | 0.99 | 0.95 | typed |
| rs8050136 | rs9923147 | 16 | 52359050 | 0.842 | C | 0.83 | 1.02 | 0.84 | imputed |
| rs8050136 | rs9923544 | 16 | 52359486 | 0.842 | C | 0.83 | 1.02 | 0.84 | imputed |
| rs8050136 | rs1558902 | 16 | 52361075 | 0.934 | A | 0.12 | 0.99 | 0.94 | imputed |
| rs8050136 | rs11075985 | 16 | 52362708 | 0.875 | A | 0.17 | 0.98 | 0.84 | imputed |
| rs8050136 | rs1121980 | 16 | 52366748 | 0.842 | A | 0.17 | 0.98 | 0.83 | typed |
| rs8050136 | rs7193144 | 16 | 52368187 | 0.967 | C | 0.12 | 1.00 | 1.0 | typed |
| rs8050136 | rs17817449 | 16 | 52370868 | 1 | G | 0.12 | 0.99 | 0.93 | imputed |
| rs8050136 | rs8043757 | 16 | 52370951 | 1 | A | 0.88 | 1.01 | 0.93 | imputed |
| rs8050136 | rs8051591 | 16 | 52374253 | 1 | A | 0.88 | 1.01 | 0.92 | imputed |
| rs8050136 | rs9935401 | 16 | 52374339 | 1 | A | 0.12 | 0.99 | 0.91 | imputed |
| rs8050136 | rs3751812 | 16 | 52375961 | 1 | G | 0.88 | 1.01 | 0.90 | imputed |
| rs8050136 | rs9936385 | 16 | 52376670 | 0.935 | C | 0.12 | 0.99 | 0.88 | imputed |
| rs8050136 | rs11075989 | 16 | 52377378 | 1 | C | 0.88 | 1.02 | 0.88 | imputed |
| rs8050136 | rs11075990 | 16 | 52377394 | 1 | A | 0.88 | 1.02 | 0.87 | imputed |
| rs8050136 | rs9939609 | 16 | 52378028 | 1 | A | 0.12 | 0.98 | 0.85 | typed |
| rs8050136 | rs7202116 | 16 | 52379116 | 0.967 | A | 0.88 | 1.01 | 0.90 | imputed |
| rs8050136 | rs7201850 | 16 | 52379363 | 0.871 | C | 0.83 | 1.02 | 0.80 | imputed |
| rs8050136 | rs7185735 | 16 | 52380152 | 0.967 | A | 0.88 | 1.01 | 0.93 | imputed |
| rs8050136 | rs9941349 | 16 | 52382989 | 0.871 | T | 0.17 | 0.98 | 0.85 | typed |
| rs8050136 | rs9931494 | 16 | 52384680 | 0.871 | C | 0.83 | 1.02 | 0.83 | imputed |
| rs8050136 | rs17817964 | 16 | 52385567 | 0.967 | C | 0.87 | 1.00 | 0.97 | imputed |
| rs8050136 | rs9930501 | 16 | 52387953 | 0.842 | A | 0.83 | 1.03 | 0.76 | imputed |
| rs8050136 | rs9930506 | 16 | 52387966 | 0.842 | G | 0.18 | 0.99 | 0.87 | typed |
| rs8050136 | rs9922708 | 16 | 52388647 | 0.842 | C | 0.83 | 1.03 | 0.75 | imputed |
| rs8050136 | rs12149832 | 16 | 52400409 | 0.934 | A | 0.13 | 0.98 | 0.83 | imputed |
| rs8050136 | rs11642841 | 16 | 52402988 | 0.87 | A | 0.04 | 1.02 | 0.90 | imputed |
| rs4523957 | rs10852932 | 17 | 2090210 | 0.835 | G | 0.76 | 0.96 | 0.61 | imputed |
| rs4523957 | rs216217 | 17 | 2091840 | 0.835 | A | 0.76 | 0.97 | 0.68 | imputed |
| rs4523957 | rs216219 | 17 | 2094041 | 0.804 | C | 0.76 | 0.97 | 0.70 | imputed |
| rs4523957 | rs216223 | 17 | 2102698 | 0.835 | C | 0.24 | 1.01 | 0.88 | typed |
| rs4523957 | rs12943566 | 17 | 2104524 | 0.835 | A | 0.24 | 1.02 | 0.84 | imputed |
| rs4523957 | rs12938295 | 17 | 2107604 | 0.835 | A | 0.24 | 1.02 | 0.84 | imputed |
| rs4523957 | rs7213232 | 17 | 2112090 | 0.835 | G | 0.24 | 1.01 | 0.86 | typed |
| rs4523957 | rs216183 | 17 | 2120673 | 0.835 | A | 0.24 | 1.02 | 0.82 | imputed |
| rs4523957 | rs11657644 | 17 | 2121960 | 0.835 | C | 0.24 | 1.02 | 0.76 | typed |
| rs4523957 | rs9905529 | 17 | 2124482 | 0.835 | C | 0.24 | 1.02 | 0.81 | imputed |
| rs4523957 | rs8065650 | 17 | 2127573 | 0.804 | T | 0.24 | 1.02 | 0.80 | typed |
| rs4523957 | rs8066372 | 17 | 2127718 | 0.804 | A | 0.76 | 0.98 | 0.79 | imputed |
| rs4523957 | rs749240 | 17 | 2132850 | 0.835 | T | 0.24 | 1.03 | 0.72 | typed |
| rs4523957 | rs216189 | 17 | 2134151 | 0.835 | G | 0.24 | 1.03 | 0.71 | typed |
| rs4523957 | rs216190 | 17 | 2134207 | 0.835 | C | 0.24 | 1.02 | 0.74 | imputed |
| rs4523957 | rs8074850 | 17 | 2134681 | 0.835 | A | 0.24 | 1.02 | 0.74 | imputed |
| rs4523957 | rs216191 | 17 | 2135389 | 0.835 | C | 0.24 | 1.03 | 0.74 | imputed |
| rs4523957 | rs403553 | 17 | 2138010 | 0.847 | G | 0.24 | 1.03 | 0.66 | typed |
| rs4523957 | rs216206 | 17 | 2142838 | 0.835 | C | 0.24 | 1.03 | 0.74 | imputed |
| rs4523957 | rs170044 | 17 | 2144252 | 0.835 | G | 0.24 | 1.03 | 0.72 | typed |
| rs4523957 | rs177567 | 17 | 2144387 | 0.835 | C | 0.24 | 1.03 | 0.73 | typed |
| rs4523957 | rs216193 | 17 | 2150203 | 0.8 | A | 0.29 | 1.03 | 0.67 | imputed |
