# Supplementary material for: Identification of New Genetic Risk Variants for Type 2 Diabetes
Source: PLoS Genet. 2010 Sep 16;6(9):e1001127. doi: 10.1371/journal.pgen.1001127 (PMC2940731; doi:10.1371/journal.pgen.1001127)
Supplement: Table S4 — Results for the four SNPs showing promising associations with T2D stratified by smoking, BMI, family history of type 2 diabetes, and age of diagnosis in the combined data. (0.07 MB DOC) [file pgen.1001127.s004.doc]

Table S4 Results for the four SNPs showing promising associations with T2D stratified by smoking, BMI, family history of type 2 diabetes, and age of diagnosis in the combined data

|  | Number | | OR (95% CI) |  | Number | | OR (95% CI) |  |  |
| --- | --- | --- | --- | --- | --- | --- | --- | --- | --- |
| SNP | Cases | Controls | Per allele | P | Cases | Controls | Per allele | P | P for interaction |
|  | Never smoking | | | | Ever smoking | | | |  |
| rs10906115 | 5461 | 7781 | 1.14 (1.08-1.21) | 7.4 x 10^-6^ | 3194 | 3622 | 1.13 (1.05-1.23) | 0.001 | 0.89 |
| rs1359790 | 5462 | 7745 | 1.19 (1.12-1.26) | 6.4 x 10^-8^ | 3194 | 3608 | 1.09 (1.00-1.19) | 0.044 | 0.11 |
| rs1436955 | 5482 | 7790 | 1.16 (1.09-1.24) | 1.3 x 10^-5^ | 3212 | 3633 | 1.05 (0.96-1.14) | 0.29 | 0.11 |
| rs10751301 | 5480 | 7788 | 1.14 (1.07-1.22) | 4.1 x 10^-5^ | 3210 | 3630 | 1.07 (0.99-1.16) | 0.11 | 0.21 |
|  | BMI <25 | | | | BMI >=25 | | | |  |
| rs10906115 | 3643 | 8119 | 1.14 (1.07-1.22) | 3.4 x 10^-5^ | 5026 | 3469 | 1.12 (1.05-1.20) | 0.74 | 0.18 |
| rs1359790 | 3648 | 8079 | 1.19 (1.11-1.28) | 5.3 x 10^-7^ | 5022 | 3458 | 1.11 (1.03-1.19) | 0.004 | 0.29 |
| rs1436955 | 3662 | 8136 | 1.16 (1.08-1.25) | 4.6 x 10^-5^ | 5046 | 3472 | 1.08 (1.00-1.17) | 0.049 | 0.22 |
| rs10751301 | 3661 | 8132 | 1.10 (1.02-1.18) | 0.009 | 5043 | 3472 | 1.12 (1.05-1.21) | 0.002 | 0.094 |
|  | With family history | | | | Without family history | | | |  |
| rs10906115 | 2733 | 1537 | 1.18 (1.06-1.31) | 0.002 | 4828 | 8070 | 1.10 (1.04-1.17) | 1.0 x 10^-4^ | 0.22 |
| rs1359790 | 2740 | 1531 | 1.17 (1.05-1.31) | 0.006 | 4832 | 8048 | 1.10 (1.03-1.18) | 2.8 x 10^-8^ | 0.37 |
| rs1436955 | 2750 | 1535 | 1.09 (0.96-1.22) | 0.18 | 4849 | 8093 | 1.11 (1.04-1.19) | 0.002 | 0.58 |
| rs10751301 | 2746 | 1537 | 1.08 (0.96-1.20) | 0.21 | 4850 | 8086 | 1.10 (1.04-1.18) | 0.003 | 0.81 |
|  | Age at diagnosis <50 vs Age at diagnosis ≥50 | | | | Age at diagnosis <55 vs Age at diagnosis ≥55 | | | |  |
| rs10906115 | 1841 | 5684 | 0.98 (0.90-1.06) | 0.63 | 2990 | 4535 | 0.98 (0.91-1.05) | 0.55 |  |
| rs1359790 | 1842 | 5687 | 0.98 (0.90-1.08) | 0.72 | 2989 | 4540 | 0.98 (0.91-1.07) | 0.69 |  |
| rs1436955 | 1852 | 5712 | 0.99 (0.90-1.09) | 0.83 | 3006 | 4558 | 0.97 (0.89-1.05) | 0.42 |  |
| rs10751301 | 1849 | 5711 | 1.09 (0.99-1.19) | 0.067 | 3001 | 4559 | 1.07 (0.99-1.16) | 0.084 |  |

Notes:

a. Analyses were adjusted for age (continuous), gender, BMI, and study site.

b. No individual data was available for Korean study, so this study was not included in these analyses.

c. For imputed SNPs, dosage data with imputation uncertainty taken into account were used to test associations.

d. No family history data were available for the SBCS or SNI.
