# Supplementary figures and images for: Identification of New Genetic Risk Variants for Type 2 Diabetes
Source: PLoS Genet. 2010 Sep 16;6(9):e1001127. doi: 10.1371/journal.pgen.1001127 (PMC2940731; doi:10.1371/journal.pgen.1001127)

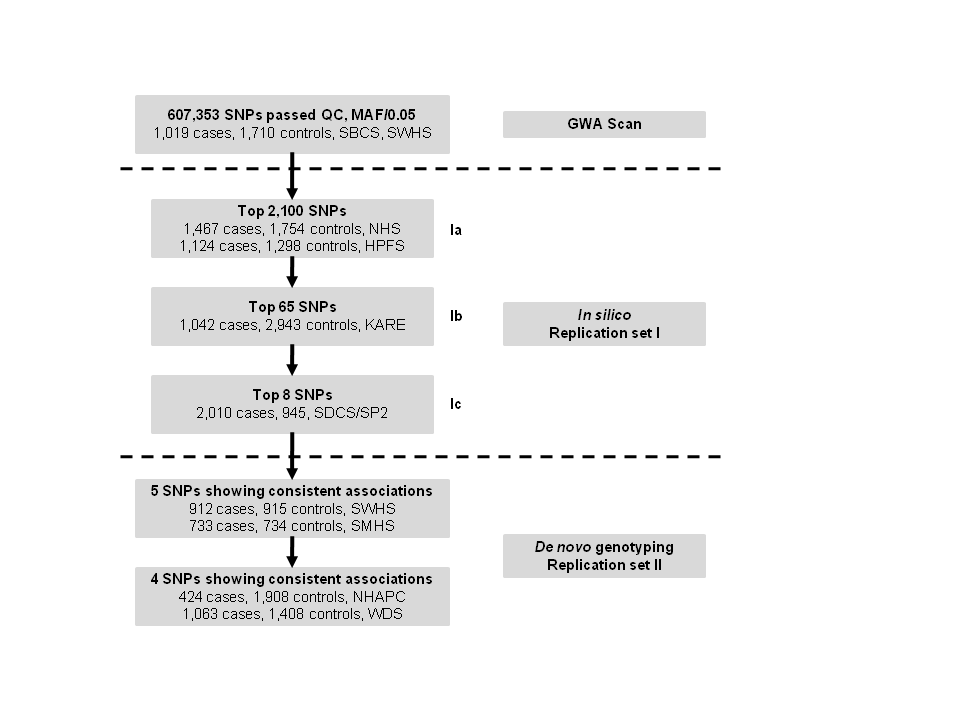

Supplement: Figure S1 — Study design. (0.09 MB TIF) [file pgen.1001127.s005.tif]

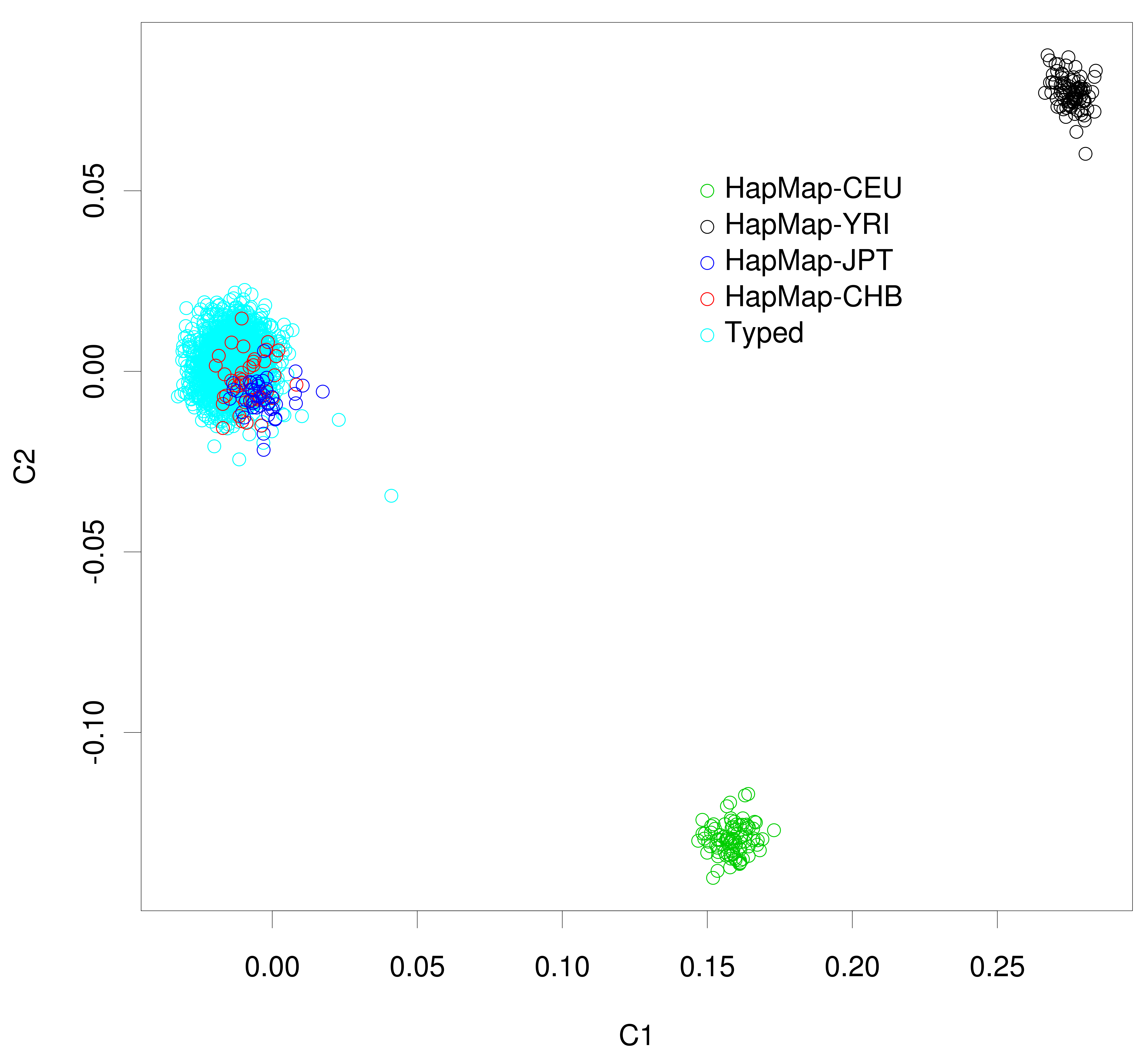

Supplement: Figure S2 — MDS analyses to confirm all subjects were Asians. (1.55 MB TIF) [file pgen.1001127.s006.tif]

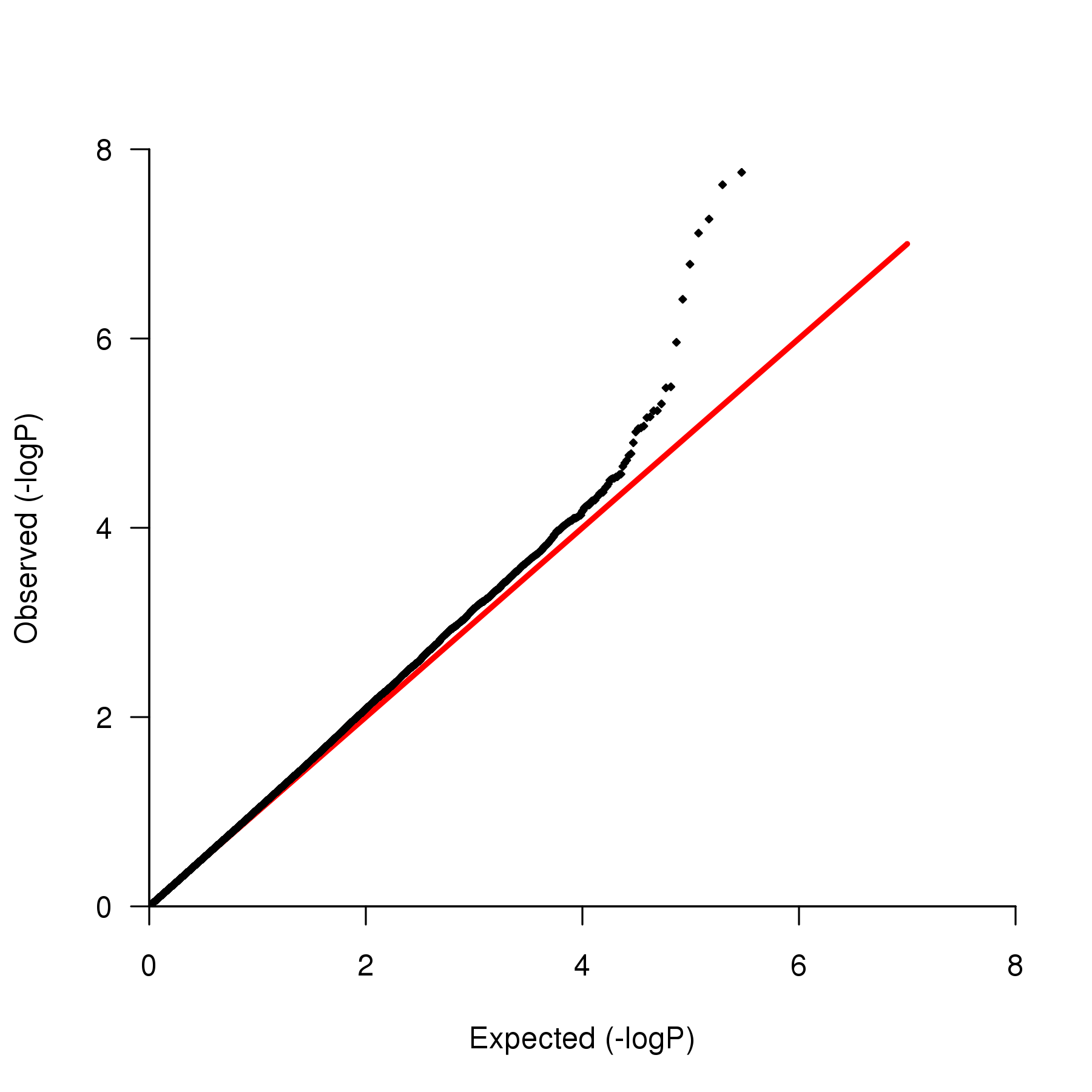

Supplement: Figure S3 — QQ Plot. (9.73 MB TIF) [file pgen.1001127.s007.tif]
