# Supplementary material for: Identification of New Genetic Risk Variants for Type 2 Diabetes
Source: PLoS Genet. 2010 Sep 16;6(9):e1001127. doi: 10.1371/journal.pgen.1001127 (PMC2940731; doi:10.1371/journal.pgen.1001127)
Supplement: Text S1 — Supplementary Methods. (0.13 MB DOC) [file pgen.1001127.s008.doc]

**Text S1 Study Participants and Methods**

**Shanghai Diabetes Genome-Wide Association Study (GWAS)**

**Discovery phase**

Study Population: Diabetes cases in the Shanghai Diabetes GWAS included 886 incident type 2 diabetes (T2D) cases identified in the Shanghai Women’s Health Study (SWHS) [1] and 133 prevalent T2D cases identified from female controls of the Shanghai Breast Cancer Study (SBCS) [2]. The SWHS is an ongoing, population-based cohort study of approximately 75,000 women recruited between 1997 and 2000 who were aged 40 to 70 years at recruitment and were permanent residents of seven communities of urban Shanghai. Details of the SWHS design and study implementation have been described elsewhere [1]. In-person interviews, anthropometrics, and blood or buccal cell sample collection were carried out by trained interviewers. Study participants are being followed through biennial in-person surveys to collect information on survival status and occurrence of cancer, diabetes, and other chronic diseases. A total of 901 women with self-reported diabetes since study enrollment that met the following criteria were included in the GWAS: 1) age ≤65, 2) using diabetes medication, 3) fasting glucose level >125 mg/dL at least twice, and 4) donated a blood sample. After quality checking using the same method described previously for the GWAS of breast cancer [2], genotyping information was available for 886 participants.

The controls used in this GWAS were shared with a GWAS that was recently completed for breast cancer and that was based primarily based on the Shanghai Breast Cancer Study (SBCS) [2]. Details of the SBCS and the breast cancer GWAS, including subject recruitment, sample collection, processing, laboratory protocols, genotyping, filtering, and data cleaning procedures have been described elsewhere [2,3]. Of the 1,938 controls included in the breast cancer GWAS that were genotyped with Affymetrix 6.0, 17 were on diabetes medication and 117 had a blood glucose level >125(mg/dL); these subjects were included as T2D cases in the current study. One of these T2D cases also participated in the SWHS. Thus, a total of 133 T2D cases identified from the SBCS controls were included in the case group of this study. Also excluded from the control group were women who had a blood glucose level between 100 and 125 mg/dL and had HbA1C>6.1 (n=54) or had no HbA1C data (n=28), women who were younger than age 35 at the time of diabetes diagnosis (n=4), and women with a self-reported history of diabetes, but who had either no information on diabetes treatment or who had a glucose level <125 mg/dL in the current study (n=8). After these exclusions, 1,710 women remained as controls for the T2D GWAS.

Genotyping methods and quality control (QC): Genomic DNA was extracted from buffy coats using a Qiagen DNA purification kit (Valencia, CA) or Puregene DNA purification kit (Minneapolis, MN) according to the manufacturers’ instructions and then used for genotyping assays. The GWAS genotyping was performed at the Vanderbilt Microarray Shared Resource (VMSR) using the Affymetrix Genome-Wide Human SNP Array 6.0 (Affy6.0) platform, following Affymetrix’s protocols. In each of the 96-well plates for Affymetrix SNP 6.0 genotyping, three positive QC samples purchased from Coriell Cell Repositories (<http://ccr.coreill.org/>) were included. The average concordance rate between the QC samples was 99.8% with median value of 100%. In addition, a series of datasets were used to assess cross-genotyping platform validation. These included the following sets of SNPs that are on the Affymetrix SNP Array 6.0 and had been genotyped previously using various platforms for a subset of subjects included in the GWAS scan: 1) 669 SNPs genotyped for 1,035 subjects by using Affymetrix Target Genotyping System; 2) 17 SNPs genotyped for 1,091 subjects by Taqman; and 3) 251 SNPs genotyped for 108 subjects by Sequenom. These three sets of SNPs served as cross-platform sample verification during the laboratory process. The mean concordance rates were 99.5%, 98.5%, and 98.9% for Affymetrix Targeted Genotyping, Taqman, and Sequenom, when compared with the Affymetrix SNP Array 6.0.

**Marker exclusion criteria:** The following quality control criteria were applied to assure the data quality of each SNP: 1) MAF <0.05; 2) call rate <95%; 3) P for HWE <0.00001 in either cases or controls or in the combined data set; 4) concordance rate <95% among duplicated QC samples; 5) significant difference in allele frequency (P<0.00001) between the 133 T2D cases from the SBCS and the 886 T2D cases from the SWHS; 6) significant difference in missing rates between cases and controls (P<0.00001). After applying the QC filter, 590,887 SNPs remained for the analyses.

**Individual exclusion criteria:** The gender of all study subjects was confirmed to be female according to the X chromosome genotyping data. Multidimensional scaling (MDS) analyses based on pairwise IBS showed that all subjects in the present study were clustered closely with HapMap Asians. We also excluded samples that had: 1) call rate <95% (n=5); 2) contaminated samples, samples with mixed-up labels, or duplicated samples (n=12); 3) first-degree relatives, such as parent-offspring and full siblings (n=4). A total of 21 subjects were excluded. Other relationships (21 half-sibling/aunt-niece pairs and 26 first-cousins/grandchild-grandparent pairs) remain in the dataset.

**Evaluation of population structure:** A set of 12,533 SNPs with a MAF ≥10% in Asians and with a distance between two adjacent SNPs of >25 kb were selected. Individual genotyping data from the present study were pooled with 210 unrelated subjects from the HapMap project. Multidimensional scaling analyses based on pairwise IBS showed that all subjects in the present study were clustered closely with HapMap Asians (Figure S2). The inflation factor
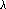
 was 1.03. Population structure was also investigated by using the principal component analysis implemented in EIGENSTRAT (http://genepath.med.harvard.edu/~reich/Software.htm). The first two principal components were included in logistic regression models for adjustment of population structures.

**Imputation:** The program MACH (<http://www.sph.umich.edu/csg/abecasis/MACH/>) was used for genotype imputation to determine the probability distribution of missing genotypes conditional on a set of known haplotypes, while simultaneously estimating the fine-scale recombination map. Imputation was based on 570,441 autosomal SNPs genotyped in Stage I that passed the QC procedure, with the phased Asian data from HapMap Phase II (release 22) as the reference. A total of 1,971,054 SNPs were successfully imputed.

**The replication sample** for the Shanghai Diabetes GWAS includes 912 incident T2D cases who were not included in the SBCS/SWHS GWAS and 915 age (+2 years) and enrollment date (+1 month)-matched controls from the SWHS. Cases were ascertained according to the above mentioned study protocol. We also included an additional 733 male incident T2D cases and 734 age and recruitment date-matched controls from the Shanghai Men’s Health Study (SMHS), an ongoing, population-based cohort study of 61,500 men who were aged 40 to 75 years at study enrollment [4]. The SMHS and SWHS are carried out in the same study communities and use virtually identical study protocols. Genotyping was performed on the iPLEX™ Sequenom MassARRAY® platform. Polymerase chain reaction (PCR) and extension primers were designed by using the MassARRAY Assay Design 3.0 software (Sequenom, Inc). PCR and extension reactions were performed according to the manufacturer’s instructions, and extension product sizes were determined by mass spectrometry using the Sequenom iPLEX system. On each 96-well plate, two negative controls (water), two blinded duplicates, and two samples from the HapMap project were included. We also included 65 subjects who had been genotyped by using the Affymetrix 6.0 chip on the Sequenom genotyping platform. The consistency rate was 100% for all SNPs for both the blinded duplicates, when compared with the HapMap data and when compared with the data from the Affymetrix 6.0. All SNPs showed very high call rates (> 99.5%).

**Replication Set I Samples**

**Nurses’ Health Study (NHS) and Health Professionals Follow-up Study (HPFS) of T2D**

Study populations: Details of the NHS and HPFS cohorts have been described previously [5,6]. Briefly, the NHS was established in 1976 when 121,700 female registered nurses aged 30 to 55 years and residing in 11 U.S. states completed a mailed questionnaire on their medical history and lifestyle. The lifestyle factors, including smoking, menopausal status, postmenopausal hormone therapy, and body weight, have been updated by validated questionnaires every 2 years. 32,826 women provided blood samples between 1989 and 1990. The HPFS is a prospective cohort study of 51,529 U.S. male health professionals aged 40 to 75 years at study initiation in 1986. Information about health and disease is assessed biennially by a self-administered questionnaire. Between 1993 and 1999, 18,159 men provided blood samples. The present study was approved by the institutional review board of the Brigham and Women’s Hospital and the Human Subjects Committee Review Board of Harvard School of Public Health.

NHS and HPFS participants for the current study were selected from among those with a blood sample using a nested case-control study design [7,8]. Diabetes cases were defined as self-reported diabetes confirmed by a validated supplementary questionnaire. For cases before 1998, diagnosis was made using criteria consistent with those proposed by the National Diabetes Data Group (NDDG) [9]. We used the American Diabetes Association diagnostic criteria for diagnosis of diabetes cases during the 1998 and 2000 cycles [10]; 98% of self-reported cases were confirmed by medical records review in both cohorts [11]. Controls were defined as those free of diabetes at the time of diagnosis of the case and remained unaffected through follow-up (2006). Although controls were originally matched per case (by gender, year of birth, month of blood collection, and fasting status), matched pairs were broken because not all subjects gave informed consent for submission of their GWAS data to dbGaP.

Genotyping and quality control: The NHS and HPFS T2D GWAS are a component of the Gene Environment-Association Studies (GENEVA) under the NIH Genes, Environment and Health Initiative (GEI). Genotyping for 2,745 patients with T2D and 3,148 healthy controls from the NHS and HPFS was performed at the Broad Center for Genotyping and Analysis using the Affymetrix Genome-Wide Human SNP Array 6.0 (Santa Clara, CA) and the Birdseed calling algorithm [12]. Genotyping data first passed Broad’s initial QC, which included SNP fingerprints for sample tracking and early detection of sample misidentification, missing call rates ≥5%, the use of a HapMap control to check genotype quality independent of study samples, and tracking of reagent and instrumental performance. Genotyping data was subsequently released for further QC to the GENEVA Coordinating Center at the University of Washington.

#### Relatedness was evaluated using pairwise identity-by-descent estimation using 80k SNPs in a method-of-moments approach implemented in PLINK software [13]. In the NHS, five pairs of duplicate samples were identified and removed. One pair of full siblings and 8 sets (6 pairs and 2 triplets) of possible first cousins were also identified. Gender was confirmed by examining the mean of the intensities of SNP probes on the X and Y chromosomes. One male sample was mis-identified as a female sample and was excluded. Twenty-seven subjects with highly variable intensity data [14] and 22 samples with a missing call rate ≥2% were also removed. In the HPFS, four pairs of duplicate samples were excluded. Three pairs of full siblings and one pair of possible cousins were identified. Six samples with evidence of contamination and 20 with highly variable intensity data were excluded, as were 13 with missing call rates ≥2%.

Population structure was investigated by principal component analysis [15]. We used a set of 12,021 SNPs selected to have very low levels of LD and to have minor allele frequencies greater than 5% in Caucasians [16]. Unrelated, genetically-inferred European-ancestry women and men passing QC were included in the current study. An additional 65 cases in the NHS and 65 cases in the HPFS suspected of having type 1 diabetes were excluded, leaving 3,221 NHS samples (1,467 cases and 1,754 controls) and 2,422 HPFS samples (1,124 cases and 1,298 controls) for the final analysis.

879,071 of the 909,622 SNP probes on the array, passed the Broad’s technical QC standards for NHS samples. 874,517 SNP probes passed this QC stage for HPFS samples. We applied the same QC parameters to both scans: excluding SNPs that were monomorphic, had a missing call rate ≥2%, more than one discordance, significant deviations from HWE (P<1×10^−4^) or a MAF <2%. Duplicate SNPs (assayed with different probes) were also removed. A total of 704,409 SNPs for NHS samples and 706,040 SNPs for HPFS samples passed QC and were included for analysis. Cluster plots of all significant SNPs considered for replication were also manually inspected for quality assurance.

**Korean Diabetes Study**

Population: Subjects for the Korean GWAS scan were recruited from the Korea Association Resource (KARE) Ansung and Ansan study cohorts (two prospective, population-based studies) described previously [17]. From a total of 10,038 KARE study participants aged 40 to 69 years at the baseline study in 2001-2002, 1,042 subjects were included as T2D cases according to the following criteria: (1) under treatment for T2D, (2) fasting plasma glucose ≥ 7 mmol/l or plasma glucose 2-h after ingestion of 75gm oral glucose load ≥ 11.1 mmol/l, and (3) age of disease onset ≥ 40 years. The inclusion criteria for non-diabetic controls (n = 2,943 ) were as follows: (1) no history of diabetes and (2) fasting plasma glucose < 5.6 mmol/l and plasma glucose 2-h after ingestion of 75gm oral glucose load < 7.8 mmol/l at both the baseline and follow up studies.

Genotyping methods and quality control: The majority of genomic DNA genotyped on the Affymetrix Genome-Wide Human SNP array 5.0 were isolated from peripheral blood drawn from the Ansung and Ansan cohort participants. Where DNA samples for genotyping were inadequate, DNA extracted from Epstein-Barr virus-immortalized lymphoblastoid cell lines (LCL) was substituted. DNA samples with low concentration were amplified before genotyping according to the manufacturer's protocol (Qiagen). Genotyping performance was identical for these three DNA sources. Bayesian Robust Linear Modeling using the Mahalanobis Distance (BRLMM) Genotyping Algorithm was used for genotype calling of 500,568 SNPs. Samples with high missing genotype call rates (>4%), high heterozygosity (>30%), or gender inconsistencies, and those obtained from individuals who had developed any kind of cancer were excluded from subsequent analyses along with related or identical individuals whose computed average pairwise identity-by-state value was higher than that estimated from first-degree relatives of Korean sib-pair samples. Samples whose genotype-inferred sex disagreed with clinical records were re-tested for sex confirmation using the SNaPshot Multiplex System (Applied Biosystems). Markers with high missing gene call rates (>5%), low MAF (<0.01), or significant deviation from Hardy-Weinberg equilibrium (*P* < 1
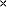
10^-6^) were excluded.

**Singapore Diabetes Study**

Population: The Singapore Diabetes Cohort Study (SDCS) comprises Chinese, Malay, and Asian-Indian men and women with T2D (http://www.med.nus.edu.sg/cof/resch_sdcs.html).  Since 2004, all individuals treated for T2D at the primary care facilities of the Singapore National Healthcare Group Polyclinics have been invited to participate in the SDCS (response rate: 91%) [18].  In this GWAS, only data from Chinese T2D cases were used.

Controls were recruited as part of the Singapore Prospective Study Program (SP2).  Individuals from 2 previous cross sectional studies, the 1992 [19] and 1998 National Health surveys [20], each representing a random sample of the Singapore population, were re-contacted between 2004 and 2007.  Subjects who were successfully re-contacted and gave informed consent answered a questionnaire and attended a clinic examination.  Fasting glucose was measured for all participants. Controls in this study included only Chinese participants with fasting plasma glucose <6.0 mmol/l, who did not report a history of physician-diagnosed T2D, and who were not taking any glucose lowering medications.

Genotyping methods and quality control: Blood-derived DNA samples of 4,693 Chinese participants of the SDCS and SP2 studies were genotyped using Illumina BeadStation and Illumina HumanHap 610 Quad, and 1Mduov3 Beadchips® (http://www.illumina.com/). Sixteen samples (8 random cases and 8 random controls) were genotyped on both Beadchips to assess genotyping quality. A total of 2,662 samples were genotyped on the 610Quad and 2,031 samples on the 1Mduov3. For each chip, a first round of clustering was performed with the proprietary clustering files from Illumina (GenCall). Samples achieving a 99% call rate were subsequently used to generate local clusterfiles (GenTrain) on which a final round of genotype calling was based. A threshold of 0.15 was implemented on the GenCall score to determine the confidence of the assigned genotypes. Samples were then removed based on the following conditions: sample call rates of less than 95%, excessive heterozygosity, cryptic relatedness by identity-by-state computation for all pairwise combinations of samples, population structure ascertainment from principal components analysis with 4 panels from the International HapMap Project (http://hapmap.ncbi.nlm.nih.gov/) and the Singapore Genome Variation Project (http://www.nus-cme.org.sg/SGVP/) with a thinned set of SNPs to reduce LD and gender discrepancies between the genetically inferred gender from Beadstudio and clinic-reported gender. We excluded 296 samples on the 610Quad and 141 samples on the 1Mduov3 in the Chinese cohort. We excluded SNPs with gross departure from HWE (p-value < 10^-4^) or that were monomorphic.

The concordance of the duplicate samples plated on different Beadarrays chips was also examined as a QC check. The average SNP concordance rate between chips for the post-QC duplicated samples was computed based on 531,805 post-QC common SNPs between chips. The mean concordance rate was 0.999. Final samples were checked for cryptic relatedness and gender discrepancies across the two chips. This led to another 140 samples excluded due to cryptic relatedness (e.g. parents-offspring, full siblings) and gender discrepancies across chips. Common SNPs across the chips were also checked for allelic differences separately in the cases and controls. A total of 97 SNPs showing significant deviation from the null were removed. In total, the post-QC dataset consisted of 1,082 cases and 1,006 controls on the 610Quad and 928 cases and 939 controls on the 1Mduov3.

**Replication Set II samples**

Replication set II samples included samples from the SWHS/SMHS (described above), Nutrition and Health of Aging Population in China (NHAPC), and Wuhan Diabetes Study (WDS).

**Nutrition and Health of Aging Population in China (NHAPC)**

The NHAPC is a population-based study comprising 3,210 individuals (1,423 men, 1,787 women) conducted among non-institutionalized Han Chinese men and women aged 50 to 70 years in Beijing and Shanghai. A multistage sampling method was used to recruit the participants. The study was conducted simultaneously in Beijing and Shanghai from March to June 2005. In each city, 2 urban districts and 1 rural district were chosen to represent people with high to low socioeconomic status. The sampling plan called for 400 participants from each urban district and 800 participants from each rural district to be selected randomly from the eligible candidates listed in the residential registration record. Potential participants had to meet the following eligibility criteria: have been a resident of the study area for at least 20 years and be free of the following conditions: 1) severe psychological disorders, physical disabilities, cancer, cardiovascular disease, Alzheimer’s disease, or dementia within the previous 6 months; and 2) currently diagnosed with tuberculosis, AIDS, and other communicable diseases. Only one person from each household was allowed to participate. It was pre-determined that at least 40% of participants would be men from each district. All participants provided written informed consent. The protocol was approved by the Institutional Review Board of the Institute for Nutritional Sciences.

An in-person interview was conducted in participants’ homes by trained physicians or public health workers from the local Centers for Disease Control and Prevention and community hospitals. Data on demographic variables, health status, health behavior, and physical activity (International Physical Activity Questionnaire, short, last-7-day format) was collected using a standardized questionnaire. The physical activity level for each individual was classified as low, moderate, or high according to the questionnaire scoring protocol (Guidelines for Data Processing and Analysis of the International Physical Activity Questionnaire [IPAQ], http://www.ipaq.ki.se/scoring.pdf). Family history of T2D was obtained for participants’ first degree relatives (e.g., participants’ parents or siblings).

All participants were invited to have a physical examination at the local health stations or the community clinics after the in-home interview. Participants were required to fast overnight. Anthropometric measurements were performed by trained medical professionals using a standardized protocol. Body weight and height were measured in light indoor clothing without shoes to the nearest 0.1 kg and 0.1 cm, respectively. Body mass index (BMI) was then calculated as weight (kg)/height (m^2^).

Glucose was measured enzymatically on an automatic analyzer (Hitachi 7080, Japan) with reagents purchased from Wako Pure Chemical Industries (Osaka, Japan). T2D was defined as fasting plasma glucose ≥ 7.0 mmol/L and/or previously diagnosed diabetes (424 T2D: 37% screening-detected, 63% previously diagnosed). Normal fasting glucose (NFG) was defined as fasting glucose <5.6 mmol/L (100mg/dL), and impaired fasting glucose (IFG) was defined as 5.6 mmol/L (100mg/dL) ≤ fasting glucose < 7.0 mmol/L (126mg/dL).

Genomic DNA was extracted from peripheral blood leucocytes by a salting-out procedure (http://www.protocol-online.org/prot/Detailed/3171.html, accessed 1 January 2009). Genotyping assays were performed with TaqMan SNP allelic discrimination by the ABI PRISM 7900HT Sequence Detection System (Applied Biosystems) according to the manufacturer’s protocol.

**Wuhan Diabetes Study (WDS)**

Population: The study includes 2,471 participants, 1,063 newly diagnosed T2D patients and 1,408 normal glucose tolerance (NGT) individuals. T2D patients were consecutively recruited from men and women attending the outpatient clinics of Department of Endocrinology, Tongji Medical College Hospital, Wuhan, China. Healthy NGT individuals were recruited from an unselected population undergoing a routine health check-up at the same hospital between December 2004 and November 2007. T2D patients met the well-established diagnostic criteria recommended by American Diabetes Association and World Health Organization incorporating both fasting plasma glucose and 2-h oral glucose tolerance test. The inclusion criteria for patients with newly diagnosed T2D were: age ≥ 30 years, no history of a diagnosis of diabetes, and no history of receiving pharmacological treatment for hyperlipidaemia or hypertension. Patients with clinically significant neurological, endocrinological or other systemic diseases, as well as acute illness or chronic inflammatory or infective diseases, were excluded from the study. All participants, including their parents and grandparents, were Han Chinese. All participants provided written, informed consent to the study and did not take any medications known to affect glucose tolerance or insulin secretion before participation.

Demographic information was collected by using a medical health questionnaire, that included questions on gender, age, height, weight, smoking and alcohol consumption (yes, ≥3 times/week; no, <3 times/week). BMI was calculated as weight divided by the square of height (kg/m^2^). After a 10-h overnight fast, all participants underwent a 75-g oral glucose tolerance test and venous blood samples were collected at 0- and 2-h for determination of fasting plasma glucose and 2-h plasma glucose. Genotyping assays were performed with TaqMan SNP allelic discrimination by the ABI PRISM 7900HT Sequence Detection System (Applied Biosystems) according to the manufacturer’s protocol.

**FIGURE LEGENDS**

Figure S1 Study design

Figure S2 QQ plot

Figure S3 MDS analyses to confirm all subjects were Asians

REFERENCES

1. Zheng W, Chow WH, Yang G, Jin F, Rothman N et al. (2005) The Shanghai Women's Health Study: rationale, study design, and baseline characteristics. Am J Epidemiol 162: 1123-1131.

2. Zheng W, Long J, Gao YT, Li C, Zheng Y et al. (2009) Genome-wide association study identifies a new breast cancer susceptibility locus at 6q25.1. Nat Genet 41: 324-328.

3. Gao YT, Shu XO, Dai Q, Potter JD, Brinton LA et al. (2000) Association of menstrual and reproductive factors with breast cancer risk: results from the Shanghai Breast Cancer Study. Int J Cancer 87: 295-300.

4. Cai H, Zheng W, Xiang YB, Xu WH, Yang G et al. (2007) Dietary patterns and their correlates among middle-aged and elderly Chinese men: a report from the Shanghai Men's Health Study. Br J Nutr 98: 1006-1013.

5. Colditz GA, Hankinson SE (2005) The Nurses' Health Study: lifestyle and health among women. Nat Rev Cancer 5: 388-396.

6. Rimm EB, Giovannucci EL, Willett WC, Colditz GA, Ascherio A et al. (1991) Prospective study of alcohol consumption and risk of coronary disease in men. Lancet 338: 464-468.

7. Qi L, Kang K, Zhang C, van Dam RM, Kraft P et al. (2008) Fat mass-and obesity-associated (FTO) gene variant is associated with obesity: longitudinal analyses in two cohort studies and functional test. Diabetes 57: 3145-3151.

8. Cornelis MC, Qi L, Zhang C, Kraft P, Manson J et al. (2009) Joint effects of common genetic variants on the risk for type 2 diabetes in U.S. men and women of European ancestry. Ann Intern Med 150: 541-550.

9. (1979) Classification and diagnosis of diabetes mellitus and other categories of glucose intolerance. National Diabetes Data Group. Diabetes 28: 1039-1057.

10. (1997) Report of the Expert Committee on the Diagnosis and Classification of Diabetes Mellitus. Diabetes Care 20: 1183-1197.

11. Hu FB, Leitzmann MF, Stampfer MJ, Colditz GA, Willett WC et al. (2001) Physical activity and television watching in relation to risk for type 2 diabetes mellitus in men. Arch Intern Med 161: 1542-1548.

12. Korn JM, Kuruvilla FG, McCarroll SA, Wysoker A, Nemesh J et al. (2008) Integrated genotype calling and association analysis of SNPs, common copy number polymorphisms and rare CNVs. Nat Genet 40: 1253-1260.

13. Purcell S, Neale B, Todd-Brown K, Thomas L, Ferreira MA et al. (2007) PLINK: a tool set for whole-genome association and population-based linkage analyses. Am J Hum Genet 81: 559-575.

14. Peiffer DA, Le JM, Steemers FJ, Chang W, Jenniges T et al. (2006) High-resolution genomic profiling of chromosomal aberrations using Infinium whole-genome genotyping. Genome Res 16: 1136-1148.

15. Patterson N, Price AL, Reich D (2006) Population structure and eigenanalysis. PLoS Genet 2: e190.

16. Yu K, Wang Z, Li Q, Wacholder S, Hunter DJ et al. (2008) Population substructure and control selection in genome-wide association studies. PLoS One 3: e2551.

17. Cho YS, Go MJ, Kim YJ, Heo JY, Oh JH et al. (2009) A large-scale genome-wide association study of Asian populations uncovers genetic factors influencing eight quantitative traits. Nat Genet 41: 527-534.

18. Ng DP, Fukushima M, Tai BC, Koh D, Leong H et al. (2008) Reduced GFR and albuminuria in Chinese type 2 diabetes mellitus patients are both independently associated with activation of the TNF-alpha system. Diabetologia 51: 2318-2324.

19. Tan CE, Emmanuel SC, Tan BY, Jacob E (1999) Prevalence of diabetes and ethnic differences in cardiovascular risk factors. The 1992 Singapore National Health Survey. Diabetes Care 22: 241-247.

20. Cutter J, Tan BY, Chew SK (2001) Levels of cardiovascular disease risk factors in Singapore following a national intervention programme. Bull World Health Organ 79: 908-915.
